# Miniature type V-F CRISPR-Cas nucleases enable targeted DNA modification in cells

**DOI:** 10.1038/s41467-021-26469-4

**Published:** 2021-10-26

**Authors:** Greta Bigelyte, Joshua K. Young, Tautvydas Karvelis, Karolina Budre, Rimante Zedaveinyte, Vesna Djukanovic, Elizabeth Van Ginkel, Sushmitha Paulraj, Stephen Gasior, Spencer Jones, Lanie Feigenbutz, Grace St. Clair, Pierluigi Barone, Jennifer Bohn, Ananta Acharya, Gina Zastrow-Hayes, Selgar Henkel-Heinecke, Arunas Silanskas, Ralf Seidel, Virginijus Siksnys

**Affiliations:** 1 Institute of Biotechnology, Life Sciences Center, Vilnius University, Vilnius, Lithuania; 2 Molecular Engineering, Corteva Agriscience™, Johnston, IA USA; 3 Institute of Experimental Physics, Leipzig University, Leipzig, Germany

**Keywords:** Biochemistry, Molecular engineering in plants, CRISPR-Cas9 genome editing

## Abstract

Class 2 CRISPR systems are exceptionally diverse, nevertheless, all share a single effector protein that contains a conserved RuvC-like nuclease domain. Interestingly, the size of these CRISPR-associated (Cas) nucleases ranges from >1000 amino acids (aa) for Cas9/Cas12a to as small as 400-600 aa for Cas12f. For in vivo genome editing applications, compact RNA-guided nucleases are desirable and would streamline cellular delivery approaches. Although miniature Cas12f effectors have been shown to cleave double-stranded DNA, targeted DNA modification in eukaryotic cells has yet to be demonstrated. Here, we biochemically characterize two miniature type V-F Cas nucleases, SpCas12f1 (497 aa) and AsCas12f1 (422 aa), and show that SpCas12f1 functions in both plant and human cells to produce targeted modifications with outcomes in plants being enhanced with short heat pulses. Our findings pave the way for the development of miniature Cas12f1-based genome editing tools.

## Abstract (web summary)

Miniature Cas12f editing systems are well suited for in vivo editing applications. Here the authors characterize the intrinsic activity of SpCas12f1 in plant and animal cells.

## Introduction

**Fig. 1:**
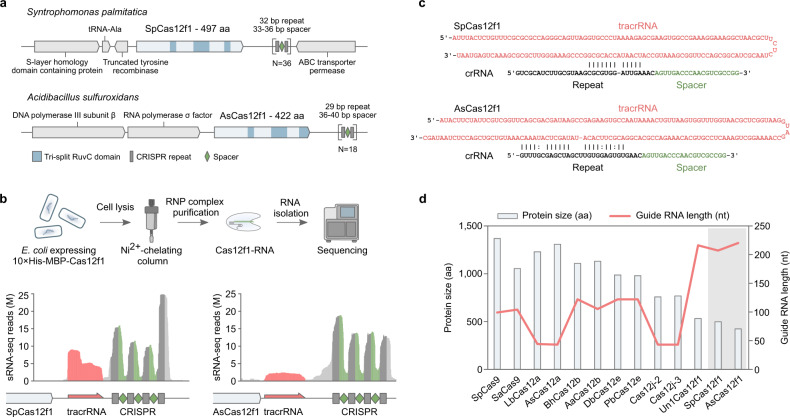
SpCas12f1 and AsCas12f1 CRISPR-Cas loci and effector complex components. **a** Schematic representation of native CRISPR-Cas loci encoding SpCas12f1 and AsCas12f1 effector proteins. **b** Workflow of the biochemical approach used to isolate and identify Cas12f1 RNP-bound RNA molecules. **c** In silico prediction of base pairing between identified tracrRNA and crRNA sequences. **d** Size comparison (aa) of Cas effector proteins (bars) and respective guide RNA length (red line) (nt) with SpCas12f1 and AsCas12f1 nucleases highlighted in the gray area.

We have recently characterized 10 exceptionally compact CRISPR (clustered regularly interspaced short palindromic repeats)-Cas12f proteins and showed that despite their miniature stature Cas12f nucleases can cleave double-stranded DNA (dsDNA) if a 5’ protospacer adjacent motif (PAM) is present in the vicinity of the target site^1^. Two systems, *Syntrophomonas palmitatica* (Sp) and *Acidibacillus sulfuroxidans* (As) (Fig. 1a), provided protection against plasmid DNA transformation in *Escherichia coli*^1^. Taken together, this indicated that they may function in heterologous cellular environments and may be harnessed as genome editing tools. Here we aim to experimentally test this hypothesis. First, we determined the key molecular and biochemical features required for SpCas12f1 and AsCas12f1 dsDNA cleavage. Subsequent leveraging of this knowledge allowed us to successfully establish genome editing with SpCas12f1 but not with AsCas12f1 in human and plant cells.

## Results

### Guide RNA (gRNA) identification

The gRNAs required for nuclease activity were experimentally characterized by sequencing Cas12f1-bound RNA species. For this, plasmids bearing CRISPR-Cas12f1 systems^1^ were modified to include a sequence that encoded a 10× histidine (His) and maltose-binding protein (MBP) (10×His:MBP) tag at the N-terminus of each Cas12f1 nuclease. After transformation and protein expression in *E. coli*, affinity tagged Cas12f1 ribonucleoprotein (RNP) complexes were pulled-down from cellular lysates and the RNA was extracted and subjected to next-generation sequencing (Fig. 1b). Subsequent analysis revealed two highly enriched RNA species (Fig. 1b). These included 40–50 nucleotide (nt) long CRISPR RNAs (crRNAs) comprising part of the repeat followed by the spacer, and a long (153 and 169 nts for SpCas12f1 and AsCas12f1, respectively) trans-activating RNA (tracrRNA) encoded between the *cas12f1* gene and the CRISPR array (Fig. 1b, c). Both tracrRNAs contained an anti-repeat region capable of base pairing with the CRISPR repeat, suggesting that the crRNA and tracrRNA may form a partial duplex (Fig. 1c). Interestingly, while SpCas12f1 and AsCas12f1 proteins are the most compact (<500 aa) class 2 CRISPR-Cas nucleases characterized to date, their gRNA length significantly exceeded that identified for other class 2 effectors (Fig. 1d)^2–12^.

### In vitro DNA target cleavage by Cas12f

**Fig. 2:**
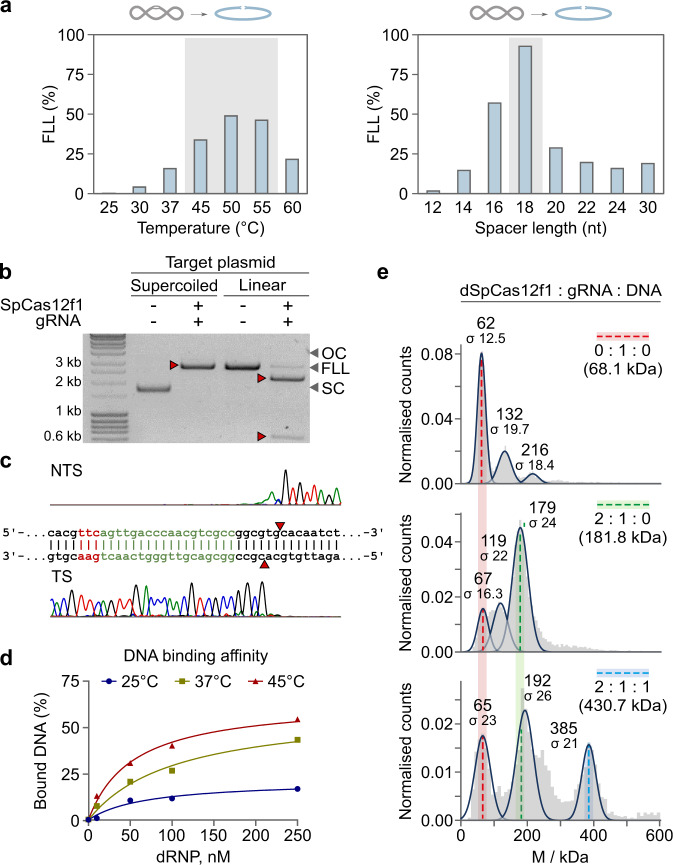
SpCas12f1 DNA cleavage and binding activity in vitro. **a** Supercoiled plasmid DNA cleavage at different temperatures (left panel) and cleavage dependence on gRNA spacer lengths at 45 °C (right panel). **b** SpCas12f1 cleavage of supercoiled and linear DNA forms in vitro. Source data are provided as a Source data file. **c** Run-off sequencing of SpCas12f1 cleavage products (cutting is centered around 22–24 bp 3’ from the PAM). NTS and TS represent non-target and target strands, respectively. **d** Dependence of dsDNA-binding efficiency on temperature. Nuclease inactivated or dead (d) SpCas12f1 (D228A) RNP was mixed with dsDNA and preincubated for 30 min at the temperatures shown. DNA binding was monitored by EMSA (electrophoretic mobility shift assay) and quantified. **e** Molecular masses of complexes measured by mass photometry. Colored dashed lines indicate the observed molecular weights for the different components: red—gRNA, green—dCas12f1–gRNA binary complex, blue—dCas12f1–gRNA–DNA ternary complex. The theoretical masses for the given complex stoichiometries are shown in brackets. For all experiments, SpCas12f1 or dSpCas12f1 RNP complexes were assembled using a gRNA with an 18 nt long spacer.

Next, we assessed the biochemical properties of SpCas12f1 and AsCas12f1 proteins. RNP complexes were assembled by mixing Cas12f1 protein with an engineered single gRNA, obtained by linking the identified crRNA and tracrRNA sequences through a tetranucleotide linker (Supplementary Table 3). Plasmid DNA digestion reactions in vitro revealed that both SpCas12f1 and AsCas12f1 cleaved dsDNA targets over a broad range of temperatures, favoring 45–55 °C, preferred different salt (NaCl) concentrations (Fig. 2a and Supplementary Fig. 1), and required spacer lengths of at least 16 nts for effective cleavage of both DNA strands (Fig. 2a and Supplementary Fig. 2). Under optimal reaction conditions, dsDNA target cleavage was next confirmed on supercoiled and linear dsDNA templates (Fig. 2b and Supplementary Fig. 3a). Run-off sequencing of the cleavage products revealed that cutting occurred between 22 and 24 bp downstream of the 5’ PAM similar to other type V effectors^1,5,8,11,12^ and was independent of spacer length (Fig. 2c and Supplementary Fig. 4). Reactions assembled with shorter synthetic double-stranded oligodeoxynucleotide substrates yielded a 5’ overhang and occurred only in the presence of a 5’ PAM (Supplementary Fig. 5), while cleavage of single-stranded DNA (ssDNA) did not require PAM recognition (Supplementary Fig. 6). Alanine substitution of the conserved aspartate residues in the RuvC active site abolished both plasmid DNA and oligodeoxynucleotide cleavage and confirmed that the RuvC domain in both proteins was responsible for DNA cleavage (Supplementary Figs. 3b and 5–7). SpCas12f1 and AsCas12f1 showed collateral nuclease activity, triggered by ssDNA and dsDNA target binding, that manifested as indiscriminate degradation of ssDNA (Supplementary Fig. 7).

### DNA target-binding activity

Optimal reaction temperatures of 45–55 °C for dsDNA cleavage by SpCas12f1 and AsCas12f1 nucleases prompted us to also investigate the effect of temperature on DNA target binding. According to gel mobility shift assays, PAM-dependent dsDNA-binding affinity increased significantly after incubation at higher temperatures (e.g., 45 °C), consistent with cleavage results (Fig. 2d and Supplementary Fig. 8). With ssDNA substrates, both nucleases robustly associated with the target DNA strand at room temperature (Supplementary Fig. 9).

### RNP complex stoichiometry

The cryo-electron microscopic (cryo-EM) structure of Un1Cas12f1^9,13^ revealed that one gRNA and two Un1Cas12f1 nucleases comprise the RNP effector complex. Here, using mass photometry^14^, we evaluated the oligomeric state of SpCas12f1 and AsCas12f1 nucleases in the apo-form, in a binary complex with its gRNA and bound to a dsDNA target in a ternary complex (Fig. 2e and Supplementary Fig. 10). In the absence of a gRNA, both nucleases were predominantly monomers, although a smaller fraction of homodimers were observed (Supplementary Fig. 10). The gRNAs were predominantly monomers (Fig. 2e and Supplementary Fig. 10). For the binary complex, the predominant species occurred at 179 and 168 kDa for SpCas12f1 and AsCas12f1, respectively, corresponding to a 2:1 Cas12f1:gRNA complex (Fig. 2e and Supplementary Fig. 10). In agreement with the cryo-EM structure of Un1Cas12f1^9,13^, the ternary SpCas12f1 and AsCas12f1:gRNA:dsDNA complexes showed a 2:1:1 stoichiometry, indicating that catalytically competent Cas12f1 complexes consisted of two nuclease subunits bound to one gRNA and a single DNA molecule (Fig. 2e and Supplementary Fig. 10).

### Genome editing in human and maize cells

**Fig. 3:**
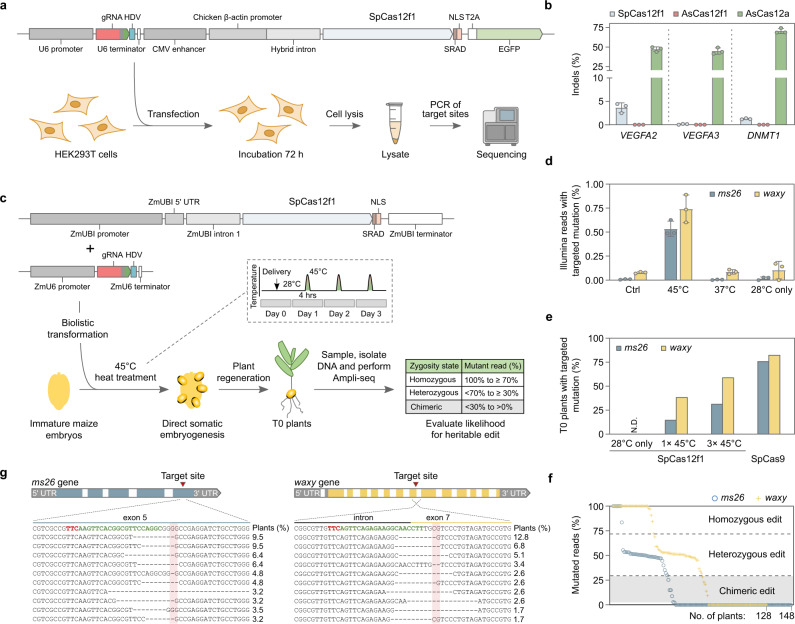
SpCas12f1 in vivo activity in human (HEK293T) and maize (*Zea mays*) cells. **a** Overview of HEK293T genome editing experimentation. Cells were transfected with a DNA expression construct encoding Cas12f1 or AsCas12a and its corresponding gRNA. **b** Frequency of indels obtained 3 days after HEK293T transfection. As a control, experiments were also conducted with *Acidaminococcus* sp. BV3L6 (As) Cas12a. Bars represent mean values with ±SD (standard deviation) error bars and dots represent data for *n* = 3 independent biological replicates. **c** Overview of *Zea mays* genome editing assay. Expression constructs were delivered using biolistic transformation and short 4 h heat treatments applied 1, 2, or 3 days after transformation. T0 plants were evaluated for the likelihood of a heritable edit. **d** Indel frequencies recovered 3 days after transformation at 28 °C or with the application of three consecutive 4 h long heat treatments at either 37 or 45 °C. Control (Ctrl) experiments were performed by omitting the gRNA expression construct from transformation. Bars represent mean values with ±SD (standard deviation) error bars and dots represent data for *n* = 3 independent biological replicates. **e** Percentage of T0 plants predicted to contain a heritable *ms26* and *waxy* targeted mutation. SpCas12f1 experiments were performed at 28 °C, with one (1 × 45 °C) or three (3 × 45 °C) 4 h 45 °C treatments. SpCas9 experiments were performed at 28 °C. **f** Predicted zygosity states of SpCas12f1 edited T0 plants. **g** Alignment of the 10 most abundant targeted indel mutations recovered in 3 × 45 °C SpCas12f1-edited T0 plants. The PAM is shown in red and protospacer target in green. The expected cut-site is indicated with red shading. Source data are provided as a Source data file.

Since both nucleases were active over a broad range of temperatures but preferentially cleaved dsDNA substrates at 45–55 °C, two different cell types were used to evaluate the genome editing potential of SpCas12f1 and AsCas12f1. These included human HEK293T cells that prefer 37 °C and *Zea mays* (maize) cells that have been shown to tolerate temperatures up to 45 °C for short periods of time^15,16^. Experiments were first performed in HEK293T cells. For this, a total of three target sites were initially selected in *VEGFA* and *DNMT1* genes next to optimal PAM sequences for SpCas12f1 (5’-TTC-3’) and AsCas12f1 (5’-YTTN-3’)^1^. Expression plasmids encoding SpCas12f1 or AsCas12f1 nuclease and its gRNA were transfected (Fig. 3a) and after 72 h cells were harvested, and each site was assayed for the presence of mutations by targeted sequencing. All three SpCas12f1 target sites tested showed evidence of DNA double-strand break (DSB) repair as revealed by mutations centered around the expected cut-site and ranged in frequency from 0.1 to 3.6% (Fig. 3b and Supplementary Fig. 11). For AsCas12f1, target cleavage could not be detected in HEK293T cells as tested here (Fig. 3b). Altogether, the similarity in biochemical profiles between SpCas12f1 and AsCas12f1 (Supplementary Fig. S1) suggests the observed difference in DNA cleavage in HEK293T cells may be due to some yet defined cellular factors.

Next, reasoning that the ambient 37 °C temperature used for culturing HEK293T cells may limit SpCas12f1 DNA target binding and cleavage, genome editing was assayed at 45 °C in *Z. mays* cells. First, targets were selected in two agronomically relevant genes, *male sterile 26* (*ms26*) and *waxy*^17,18^, and nuclease and gRNA expression constructs built (Fig. 3c). Next, 24 h after biolistic transformation, immature embryos were incubated at 45 °C for 4 h once per day for a total of 3 days (Fig. 3c). As controls, embryos were also incubated at 28 °C and at 37 °C. The timing and length of the 37 °C incubations were as described for the 45 °C treatments while the immature embryos subjected to 28 °C were maintained at this temperature for the duration of the experiment. After the last heat treatment, embryos were harvested and target regions were deep sequenced. Both *ms26* and *waxy* sites yielded evidence of targeted mutagenesis but only in the treatments incubated at 45 °C (Fig. 3d and Supplementary Fig. 12).

To corroborate these findings, transformation was repeated with either one or three 45 °C incubations and T0 plants were regenerated. As a control, experiments were also performed without a heat treatment (28 °C). Since maize is diploid, plants with ~50% and ~100% mutant reads could be assumed to be heterozygous and homozygous, respectively, and contain a germ line genomic change that would be heritable in the next generation as described earlier^19–22^ (Fig. 3c). Like observed just 3 days after transformation, analysis of T0 plants showed the presence of DNA sequence alterations but only after at least one 45 °C heat treatment (Fig. 3e). Here 38% of the SpCas12f1-transformed plants contained a targeted *waxy* mutation classified as either heterozygous or homozygous after a single 45 °C incubation, which rose to 59% after three heat treatments (Fig. 3e, f and Supplementary Data 1). At the *ms26* site, 14% of the T0 plants were observed to be heterozygous or homozygous for targeted alterations after a single 45 °C treatment and this increased to 31% after three consecutive heat treatments (Fig. 3e, f and Supplementary Data 2). Similar to human cell experiments, SpCas12f1-targeted alterations consisted predominately of deletions that originated near or spanned the expected cut-site (Fig. 3g). Experiments were also conducted using *Streptococcus pyogenes* (Sp) Cas9 and gRNAs programmed to target regions overlapping with SpCas12f1 *ms26* and *waxy* sites (Supplementary Fig. 13). When averaged across both targets, the editing efficiencies of SpCas12f1 using three 4 h heat treatments were half to two-thirds of those produced with a constitutively active SpCas9, altogether showing that SpCas12f1 activity could be rescued by increasing cellular temperatures to ~45 °C (Fig. 3e).

### Off-target genome editing by SpCas12f1

Genomic off-target cleavage was next evaluated for SpCas12f1 in T0 *Z. mays* plants. Putative off-sites were predicted bioinformatically and then examined for evidence of nuclease-induced insertion or deletion mutations using next-generation sequencing. All genomic sites that differed by one, two, or three single-nucleotide polymorphisms (SNPs) in the gRNA target or up to two insertions or deletions or bulges between the gRNA and DNA target in combination with one or two SNPs were assayed (Supplementary Data 3). For the large number of predicted off-targets beyond this set that differed by as many as four SNPs and two bulges (6 differences in total), 69 and 75% of the SpCas12f1 *ms26* and *waxy* sites, respectively, were assayed when averaged across target sites (Supplementary Data 3). In all, no evidence of off-target genome editing was identified at the locations examined (Supplementary Data 4).

## Discussion

In this work, we characterized two exceptionally compact Cas12f1 nucleases, SpCas12f1 and AsCas12f1, and to our knowledge, provide the first demonstration of SpCas12f1-mediated genome editing in eukaryotic cells. Furthermore, we show that Cas12f1 nucleases share a set of common features: (i) compact size compared to other Cas12 nucleases; (ii) remarkably long tracrRNAs; (iii) preferential dsDNA target binding and cleavage at higher temperatures (45–55 °C); (iv) nondiscriminatory ssDNA degradation upon target binding; (v) dimerization upon binding of a single copy of gRNA. While some of these attributes (iii and iv) are shared with other class 2 effectors, others are not (i, ii, v)^5,6,11,12,23–25^. Altogether, these unique properties bring additional flexibility to the CRISPR-Cas toolbox. This includes temperature-dependent dsDNA target recognition and collateral ssDNA nuclease activity that may be advantageous in nucleic acid detection platforms when being simplified as “one pot” reactions by combining both isothermal amplification and Cas12-based detection^26–29^. Moreover, sensitivity to temperature may be used to precisely regulate activity at dsDNA targets in plants shown to tolerate or be acclimated to elevated temperatures^15,30–36^, ultimately, reducing the potential for off-target effects and allowing activity to be controlled in a spatial–temporal fashion without the need for inducible promoters. In agreement with this, our initial assessment of SpCas12f1 specificity yielded no evidence of off-site mutagenesis, although future studies with other methods and additional target sites are warranted. Finally, the small size and self-dimerization of Cas12f1 enzymes provide an advantage for viral-based delivery since the exceptionally compact transcript size of the nuclease would help to overcome viral genome packaging constraints.

## Methods

### Modification of CRISPR-Cas12f1 systems for gRNA pull-down

Plasmid-borne SpCas12f1 and AsCas12f1 CRISPR systems described earlier^1^ were engineered to also encode a 10×His:MBP tag fused to N-terminus of the *cas12f1* gene. Additionally, a sequence encoding the tobacco etch virus (TEV) protease recognition sequence (ENLYFQS) was also included. The SpCas12f1 plasmid was digested with EcoNI and NcoI restriction enzymes (NEB) and the backbone was isolated by agarose gel purification (Qiagen). Next, a synthesized DNA fragment (Genscript) containing a 5’ EcoNI restriction site, T7 promoter, lac operator, and ribozyme-binding sequence in addition to the sequence encoding the 10×His:MBP:TEV tag followed by an inverted BbsI site incorporating a sequence that upon digestion would yield a compatible NcoI overhang was digested with EcoNI and BbsI and column purified (Qiagen). The two purified fragments were then joined using T4 DNA ligase (NEB), transformed into One Shot TOP10 *E. coli* cells (Invitrogen), and constructs confirmed by Sanger sequencing. For AsCas12f1, a similar strategy was used except EcoNI and AvaI restriction enzymes (NEB) were used. Links to the plasmid sequences (pMBP-SpCas12f1 and pMBP-AsCas12f1) are provided in Supplementary Table 1.

### Expression and purification of Cas12f1-RNA complexes and Cas12f1 proteins

To obtain Cas12f1-RNA complexes, pMBP-SpCas12f1 and pMBP-AsCas12f1 plasmid-borne CRISPR systems encoding both nuclease and gRNA were transformed into *E. coli* cells (Arctic Express (DE3)). Cultures were grown in LB broth supplemented with ampicillin (100 µg/ml) and gentamicin (10 µg/ml) at 37 °C to an OD_600_ of 0.6–0.8. At this point, the temperature was decreased to 16 °C and expression induced with 0.5 mM IPTG. After 16 h, cells were pelleted, re-suspended in loading buffer (20 mM Tris-HCl (pH 8.0 at 25 °C), 250 mM NaCl, 5 mM 2-mercaptoethanol, 25 mM imidazole, 2 mM PMSF, 5% (v/v) glycerol), and disrupted by sonication. After removing cell debris by centrifugation, the supernatant was loaded on Ni^2+^-charged HiTrap chelating HP column (GE Healthcare) and eluted with a linear gradient of increasing imidazole concentration (from 25 to 500 mM) in 20 mM Tris-HCl (pH 8.0 at 25 °C), 250 mM NaCl, 5 mM 2-mercaptoethanol, and 5% (v/v) glycerol. The fractions with Cas12f1-RNA complexes were then dialyzed against 20 mM Tris-HCl (pH 8.0 at 25 °C), 250 mM NaCl, 2 mM dithiothreitol (DTT), and 50% (v/v) glycerol and stored at −20 °C.

SpCas12f1 and AsCas12f1 proteins (without gRNAs) were also expressed and purified using pMBP-SpCas12f1 and pMBP-AsCas12f1. For experimentation requiring dead (d) or nuclease-inactivated Cas12f1 protein, pMBP-SpCas12f1 and pMBP-AsCas12f1 were further modified introducing D228A and D225A encoding codons into SpCas12f1 and AsCas12f1 genes, respectively, using the Phusion Site-Directed Mutagenesis Kit (Thermo Fisher Scientific). *E. coli* cells were grown in LB broth supplemented with ampicillin (100 µg/ml) and gentamicin (10 µg/ml) at 37 °C. After culturing to an OD_600_ of 0.6–0.8, temperature was decreased to 16 °C and protein expression induced with 0.5 mM IPTG. After 16 h, cells were pelleted, re-suspended in loading buffer (20 mM Tris-HCl (pH 8.0 at 25 °C), 1.5 M NaCl, 5 mM 2-mercaptoethanol, 25 mM imidazole, 2 mM PMSF, 5% (v/v) glycerol), and disrupted by sonication. Cell debris was removed by centrifugation. The supernatant was loaded on Ni^2+^-charged HiTrap chelating HP column (GE Healthcare) and eluted with a linear gradient of increasing imidazole concentration (from 25 to 500 mM) in 20 mM Tris-HCl (pH 8.0 at 25 °C), 0.5 M NaCl, and 5 mM 2-mercaptoethanol. The fractions containing Cas12f1 protein were pooled and subsequently loaded on HiTrap heparin HP column (GE Healthcare). Linear gradient of increasing NaCl concentration (from 0.2 to 1.0 M) was used for elution. The fractions containing the protein of interest were pooled and the 10×His:MBP:TEV tag was cleaved by incubating overnight with TEV protease at 4 °C. To remove the cleaved 10×His:MBP:TEV tag and TEV protease, reaction mixtures were loaded onto a HiTrap heparin HP 5 column (GE Healthcare), and a linear gradient of increasing NaCl concentration (from 0.2 to 1.0 M) was used for elution. The collected fractions with Cas12f1 were then dialyzed against 20 mM Tris-HCl (pH 8.0 at 25 °C), 500 mM NaCl, 2 mM DTT, and 50% (v/v) glycerol and stored at −20 °C. The sequences of the Cas12f1 proteins are listed in Supplementary Table 2.

### RNA purification from Cas12f1-RNA complex

To isolate Cas12f1-bound RNA species, SpCas12f1 and AsCas12f1 RNP complexes (250 μl) were incubated with 5 μl (20 mg/ml) of Proteinase K (Thermo Fisher Scientific) for 45 min at 37 °C in 1 ml of 10 mM Tris-HCl (pH 7.5 at 37 °C), 1 mM EDTA, 1 mM DTT, 100 mM NaCl, and 5 mM MgCl_2_ buffer. Furthermore, DNA was removed by incubation for 45 min at 37 °C with 10 μl of DNase I (Thermo Fisher Scientific). The RNA was purified using a GeneJet PCR Purification column (Thermo Fisher Scientific) and eluted in nuclease-free water. RNA concentration and purity were measured by NanoDrop spectrophotometer and RNA integrity was visualized by separating reaction products on TBE-Urea (8 M) 15% denaturing polyacrylamide gel with 0.5×TBE (Tris-borate-EDTA) buffer (Thermo Fisher Scientific) and staining with SYBR Gold (Thermo Fisher Scientific).

### RNA sequencing and analysis

Purified RNA was prepared for sequencing using a TruSeq Small RNA Library Preparation Kit (Illumina) according to the manufacturer’s instruction except that an expanded size selection was performed allowing RNA species ~30–300 nts in length to be captured. After library preparation, 150 nt paired-end sequencing was performed on a MiSeq System (Illumina). The resulting data were post-processed trimming to a Phred quality score of 13, adapters hard-clipped using Cutadapt v2.10, and mapped to the reference using Bowtie2 v2.4.2^37^. Coverage data were then viewed in IGV^38^ and crRNA and tracrRNA species were identified from the resulting read pileups.

### RNA synthesis

Templates for T7 transcription of Cas12f1 single gRNAs were generated by PCR using overlapping oligonucleotides, altogether containing a T7 promoter followed by the gRNA sequence. RNAs were produced by in vitro transcription using the TranscriptAid T7 High Yield Transcription Kit (Thermo Fisher Scientific) and purified using the GeneJET RNA Purification Kit (Thermo Fisher Scientific). Sequences of the gRNAs used in our study are available in Supplementary Table 3.

### DNA substrate generation

Complementary oligonucleotides (Metabion) containing target and PAM sequences were annealed and cloned into pUC18 plasmid over HindIII (Thermo Fisher Scientific) and EcoRI (Thermo Fisher Scientific) restriction sites. The links to the plasmid sequences are provided in Supplementary Table 1.

The 5’-ends of oligonucleotides were first radiolabeled using T4 PNK (Thermo Fisher Scientific) and [γ-^32^P]ATP (PerkinElmer). Then DNA substrates were generated by annealing two oligonucleotides with complementary sequences of whom one already had a radioactive label introduced at the 5’-end. Annealing was performed at 95 °C following slow cooling to room temperature. The sequences of the oligoduplexes are provided in Supplementary Table 4.

### Cas12f1-gRNA complex assembly for in vitro DNA cleavage

In all, 1 µM of purified Cas12f1 protein was combined with its corresponding gRNA in 1:1 molar ratio in complex assembly buffer (10 mM Tris-HCl (pH 7.5 at 37 °C), 100 mM NaCl, 1 mM EDTA, 1 mM DTT) and allowed to incubate at 37 °C for 30 min.

### DNA cleavage assays

Reaction mixtures of 3 nM plasmid DNA, 100 nM Cas12f1 RNP complex in 10 mM Tris-HCl (pH 7.5 at 37 °C), 1 mM EDTA, 1 mM DTT, 10 mM MgCl_2_, and 200 or 100 mM NaCl buffer for SpCas12f1 and AsCas12f1, respectively, were incubated at 45 °C or as specified. The reaction was initiated by addition of Cas12f1 RNP complexes and was quenched at timed intervals (60 min if not indicated differently) by mixing with 3× loading dye solution (0.01% Bromophenol Blue and 75 mM EDTA in 50% (v/v) glycerol)). Reaction products were analyzed by agarose gel electrophoresis and ethidium bromide staining.

Reactions with oligoduplexes or ssDNA oligonucleotides were typically carried out by mixing labeled DNA samples with Cas12f1 RNP complex and incubating at 45 °C. Reaction mixtures contained 1 nM labeled duplex, 100 nM Cas12f1 RNP complex, 10 mM Tris-HCl (pH 7.5 at 37 °C), 1 mM EDTA, 1 mM DTT, 10 mM MgCl_2_, and 200 or 100 mM NaCl for SpCas12f1 and AsCas12f1, respectively, in a 100 µl final volume. Aliquots of 6 μl were removed from the reaction mixture at timed intervals (0, 5, 15, 30, and 60 min for SpCas12f1 or 0, 1, 5, 15, and 30 min for AsCas12f1) and quenched with 10 μl of a loading dye (95% (v/v) formamide, 0.01% Bromophenol Blue, and 25 mM EDTA). Reaction products were analyzed by denaturing gel electrophoresis (20% polyacrylamide containing 8.5 M urea in 0.5× TBE buffer), which were dried and visualized by phosphorimaging.

### DNA-binding assay

Binding assays were performed by incubating different amounts of Cas12f1 RNP complexes (0, 10, 50, 100, and 250 nM) with 1 nM of ^32^P-5′-labeled ssDNA or dsDNA substrates (Supplementary Table 4) in binding buffer (40 mM Tris, 20 mM acetic acid (pH 8.4 at 25 °C), 1 mM EDTA, 0.1 mg/ml bovine serum albumin, 10% (v/v) glycerol, and 5 mM Mg(C_2_H_3_O_2_)_2_). All reactions were incubated for 30 min at room temperature (or as indicated) prior to electrophoresis on a native 8% (w/v) polyacrylamide gel. Electrophoresis was carried out at room temperature for 3 h at 110 V using 40 mM Tris, 20 mM acetic acid (pH 8.4 at 25 °C), 1 mM EDTA, and 5 mM Mg(C_2_H_3_O_2_)_2_ as the running buffer. Gels were dried and visualized by phosphorimaging.

### Molecular weight measurements by mass photometry

Measurements were performed on an OneMP mass photometer (Refeyn Ltd). To prepare the measurements, coverslips (No. 1.5 H, 24 × 50 mm, Marienfeld) were cleaned by sequential sonication for 5 min in Milli-Q-water, isopropanol and Milli-Q-water. Coverslips were then dried using a clean stream of nitrogen. Measurement stock solutions of Cas12f1 RNP complex were prepared freshly before each measurement by mixing Cas12f1 protein (1 µM) and gRNA (500 nM) in complex assembly buffer (10 mM Tris-HCl (pH 7.5 at 37 °C), 100 mM NaCl, 1 mM EDTA, 1 mM DTT) followed by incubation at 37 °C for 30 min. To prepare measurement stock solutions for pure Cas12f1 protein, gRNA and DNA samples of the respective stock solutions were diluted to 500 nM concentration in complex assembly buffer and incubated for 30 min at 37 °C. For DNA-binding experiments, 200 nM Cas12f1 RNP complex and 25 nM DNA were mixed in binding buffer (40 mM Tris-HAc (pH 8.4 at 25 °C), 5 mM Mg(CH_3_COO)_2_) and incubated for 30 min at 45 °C. After incubation, all samples were diluted by 1:10 in the respective sample buffer just before the measurement. Prior to the measurements, a cleaned coverslip was mounted onto the mass photometer and a gasket (CultureWell™ Reusable Gasket, Grace Bio-Labs) was placed on top. A gasket well was filled with 10 µl of the corresponding sample buffer, 10 µl of the diluted sample were added, and the adsorption of biomolecules was monitored for 120 s using the AcquireMP software (Refeyn Ltd, Version 2.3.0). For converting the measured ratiometric contrast into molecular mass, Un1Cas12f1 and its oligomers ranging from 60 to 250 kDa (monomer to tetramer) were used for calibration. All mass photometry movies were analyzed using DiscoverMP (Refeyn Ltd, Version 2.3.0). All samples were measured in triplicates.

### M13 cleavage assay

M13 ssDNA cleavage reactions were initiated by mixing M13 ssDNA (New England Biolabs) with/or without DNA activator and Cas12f1 RNP complex at 45 °C. In all, 10 mM Tris-HCl (pH 7.5 at 37 °C), 1 mM EDTA, 1 mM DTT, 10 mM MgCl_2_, and 200 or 100 mM NaCl buffers were used, respectively, for SpCas12f1 and AsCas12f1. The final reaction mixture consisted of 3 nM M13 ssDNA, 100 nM ssDNA or dsDNA activator, or no activator and 100 nM Cas12f1 RNP. After initiating the reaction by adding Cas12f1 RNP, the samples were collected at timed intervals (0, 5, 15, 30, 60 min) by mixing with 3× loading dye solution (0.01% Bromophenol Blue and 75 mM EDTA in 50% (v/v) glycerol). Reaction products were separated on an agarose gel and stained with SYBR Gold (Thermo Fisher Scientific). The sequences of the activators are listed in Supplementary Table 4.

### Human cell culture and transfection

HEK293T cells were purchased from ATCC (catalog number CRL-3216) and cultivated using Dulbecco’s Modified Eagle Medium (DMEM) supplied with 10% fetal bovine serum, penicillin (100 U/ml), and streptomycin (100 µg/ml) (Thermo Fisher Scientific). Cells were first seeded in a 24-well plate at a density of 1.4 × 10^5^ cells/well. After approximately 1 day of growth, a transfection mixture was prepared by diluting 1 µg of nuclease and its gRNA-containing plasmid (listed in Supplementary Table 1) in 100 µl serum-free DMEM and 2 µl of TurboFect transfection reagent was added (Thermo Fisher Scientific). After a 15 min incubation at room temperature, the transfection mixture was then added dropwise to each well containing the prepared cells. Transfected cells were then grown for 72 h at 37 °C and 5% CO_2_.

### *Z. mays* transformation

First, 0.6 µM (average size) gold particles were coated with SpCas12f1 expression cassettes (Supplementary Table 1) using TransIT-2020, pelleted by centrifugation, washed with ethanol, and resuspended using sonication. Ten microliters of the DNA-linked gold particles were then loaded onto a microcarrier and allowed to air dry. Using a PDS-1000/He gun (Bio-Rad), particles were next bombarded into 9–10-day-old immature maize embryos (genotype PH1V69) with a 425 lb/in^2^ rupture disc. For transient assays, a gene encoding a yellow fluorescent protein, ZsYELLOW1 N1^39^, was also delivered to aid in the selection of evenly transformed embryos 3 days after transformation. To produce T0 plants, post-bombardment culture, selection, and plant regeneration were performed using methods described previously^40^ except that *bbm* and *wus2* genes were expressed with non-constitutive promoters, maize phospholipid transferase protein (Zm-PLTP), and maize auxin-inducible (Zm-Axig1) promoters, respectively^41^.

### Human and *Z. mays* genome editing assay

Transfected HEK293T cells were collected by trypsinization and their genomic DNA was extracted using QuickExtract solution (Lucigen). For transient *Z. mays* assays, immature embryos were harvested 3 days post transformation, lyophilized, finely ground, and their total DNA extracted using the Synergy 2.0 Plant DNA Extraction Kit (Ops Diagnostics). To ensure chimeric and germ line edits could be distinguished in T0 plants, two leaf punches were taken from different leaves (V2 or V3). The fresh tissue was then ground, and DNA was extracted using PB buffer (Qiagen) in combination with a glass fiber 96-well microfilter plate (Agilent). PCR was then performed in two rounds to amplify the DNA region surrounding each target site and add on the sequences required for Illumina sequencing and indexing^20,42^. Briefly, 1–4 µl of DNA (10–200 ng) was used in a primary PCR with primers specific to the genomic locus that were 5’ tailed with Illumina sequences in a final volume of 20–50 µl (Supplementary Table S5). To ensure a balanced read composition within the initial cycles of sequencing, a mixture of four forward primers were used (see F1–F4 in Supplementary Table S5). Each of these primers was identical except for a 6 nt region immediately 3’ of the Illumina sequencing primer-binding site (Supplementary Table S5). Primary PCR was followed by a second round of PCR using 1 µl of the initial reaction as a template and primers specific to the Illumina sequences added in the primary PCR that also encoded the remaining sequences needed for Illumina bridge amplification, sequencing, and data deconvolution (in a 20–50 µl final volume). All primers and targets can be found in Supplementary Tables 5 and 6, respectively. Both rounds of PCR were allowed to proceed for 20 cycles and were carried out using NEBNext Q5 Hot Start HiFi PCR Master Mix (NEB), Phusion High-Fidelity PCR Master Mix with GC Buffer (ThermoFisher Scientific), or Platinum SuperFi II Master Mix with Green Dye (ThermoFisher Scientific) according to the manufacturer’s instruction. After PCR, 5–10 µl were separated on a 1–2% agarose gel, stained with RedSafe (iNtRON) or ethidium bromide (Sigma), and visualized relative to DNA molecular weight standards to be the correct size. DNA was then purified using a Monarch PCR purification column (NEB) or Zymoclean Gel DNA Recovery Kit (Zymo Research), combined in an equimolar fashion, and sequenced on a MiSeq System (Illumina) with custom sequence primers, one for the amplicon and the second for the index (Supplementary Table 5). Sequences were trimmed to a Phred quality score of 13 and evaluated using a custom script^43^ for detection of insertion or deletion (indel) mutations that occurred within the expected cut-site. To be considered as true evidence of DSB repair, indel types were grouped, counted, and required to be at least 30 times greater in frequency than that found in the negative controls. The frequency of mutant reads was calculated by dividing the total number of mutant reads by the total number of wild-type reads. Mutant reads were visualized by aligning them against the wild-type reference highlighting the differences in contrasting colors. Percentage of edited plants (Fig. 3g) was calculated by dividing the number of plants with the specific mutation by the total number of plants with targeted modification.

### SpCas12f1 specificity

Potential off-target sites were identified using Cas-OFFinder^44^. Molecular inversion probes (MIPs) were designed within a 50–400 bp window spanning all sites with up to four mismatches and two bulges and synthesized as a high-density pool from LC Sciences (Houston, TX). Altogether, 20 T0 plants that contained an on-target alteration classified as either heterozygous or homozygous, were sampled. This included five plants from the *ms26* 1 × 45 °C, *ms26* 3 × 45 °C, *waxy* 1 × 45 °C, and *waxy* 3 × 45 °C experiments. For use as a negative control, seven wild-type PH1V69 plants were also sampled. MIP targeting and sequence pools were prepared, and indexed amplicons were generated as described earlier^45^. Following Ampure XP PCR purification (Beckman Coulter Inc.), libraries were sequenced on an Illumina NextSeq, producing 150 nt paired end reads. If needed, targeted next-generation sequencing was used to supplement MIP sequence coverage for sites more closely related to the on-target (up to three SNPs and two SNPs in combination with two bulges). After sequencing, low-quality reads (phred quality score <30) were discarded and mapped to the PH1V69 reference genome with Bowtie2^37^. For a given target site to be considered, at least five reads were required to be uniquely mapped to the locus harboring the off-site. The alignment files were next processed with a custom python script to characterize the edits^46^. Alterations that occurred in a 12 base pair window centered over the expected cleavage site were flagged and examined further. If the total number of mutant reads were >20% and not present in negative controls, the site was designated as a putative edit and manually verified by visualizing the reads with IGV^38^. All MIP sequences, targeted sequencing primers, and associated amplicons can be found in Supplementary Data 4.

### Statistics and reproducibility

All statistical analyses were performed using GraphPad Prism (v.8.4.3). The exact replication numbers are indicated in the figure legends. The findings in all the figures of the gel images were successfully reproduced in similar experimental conditions at least three times independently.

### Reporting summary

Further information on research design is available in the Nature Research Reporting Summary linked to this article.

## Supplementary information

Supplementary Information

Supplementary Data 1

Supplementary Data 2

Supplementary Data 3

Supplementary Data 4

Description of Additional Supplementary Files

Reporting Summary

## Data Availability

All data are available in the manuscript or the Supplementary Material. Illumina sequence data generated in this study have been deposited in the NCBI Sequence Read Archive database under BioProject ID PRJNA728251. Subject to any third party rights, the genomic reference for PH1V69 may be made available under an applicable material transfer agreement to academic investigators for academic, noncommercial research directed to the results in this manuscript. Source data are provided with this paper.

## Source data

Source data

## Code availability

Scripts used to analyze deep sequencing data are available on GitHub^43,46^.
