# Supplementary material for: Miniature type V-F CRISPR-Cas nucleases enable targeted DNA modification in cells
Source: Nat Commun. 2021 Oct 26;12:6191. doi: 10.1038/s41467-021-26469-4 (PMC8548392; doi:10.1038/s41467-021-26469-4)
Supplement: Supplementary file 1 — Supplementary Information [file 41467_2021_26469_MOESM1_ESM.pdf]

Supplementary information

- **Supplementary Fig. 1 |** SpCas12f1 and AsCas12f1 optimal plasmid DNA cleavage conditions in vitro.
- **Supplementary Fig. 2 |** Optimal Cas12f1 gRNA spacer length.
- **Supplementary Fig. 3 |** Cas12f1 cleavage of double-stranded DNA targets.
- **Supplementary Fig. 4 |** Run-off sequencing of Cas12f1 cleaved plasmid DNA.
- **Supplementary Fig. 5 |** Oligoduplex cleavage by Cas12f1 RNP complexes.
- **Supplementary Fig. 6 |** ssDNA cleavage by Cas12f1 RNP complexes.
- **Supplementary Fig. 7 |** Collateral ssDNA cleavage activity of Cas12f1 RNP complexes.
- **Supplementary Fig. 8 |** Temperature dependent dsDNA binding by Cas12f1 RNP complexes.
- **Supplementary Fig. 9 |** ds and ssDNA binding activity of Cas12f1 RNP complexes.
- **Supplementary Fig. 10 |** Molecular weight measurements of Cas12f1 protein and RNP complexes using mass photometry.
- **Supplementary Fig. 11 |** SpCas12f1 genome editing in HEK293T cells.
- **Supplementary Fig. 12 |** SpCas12f1 transient editing in *Zea mays* cells.
- **Supplementary Fig. 13 |** Overlap of SpCas12f1 and SpCas9 *ms26* and *waxy* targets.
- **Supplementary Table 1 |** Plasmids used in this study.
- **Supplementary Table 2 |** Sequences of the proteins used in this study.
- **Supplementary Table 3 |** RNAs used in this study.
- **Supplementary Table 4 |** Oligonucleotides and DNA activators used for different cleavage assays in this study.
- **Supplementary Table 5 |** Primers used for human and maize cells genome editing assay.
- **Supplementary Table 6 |** gRNA targets used for human and maize cells genome editing.

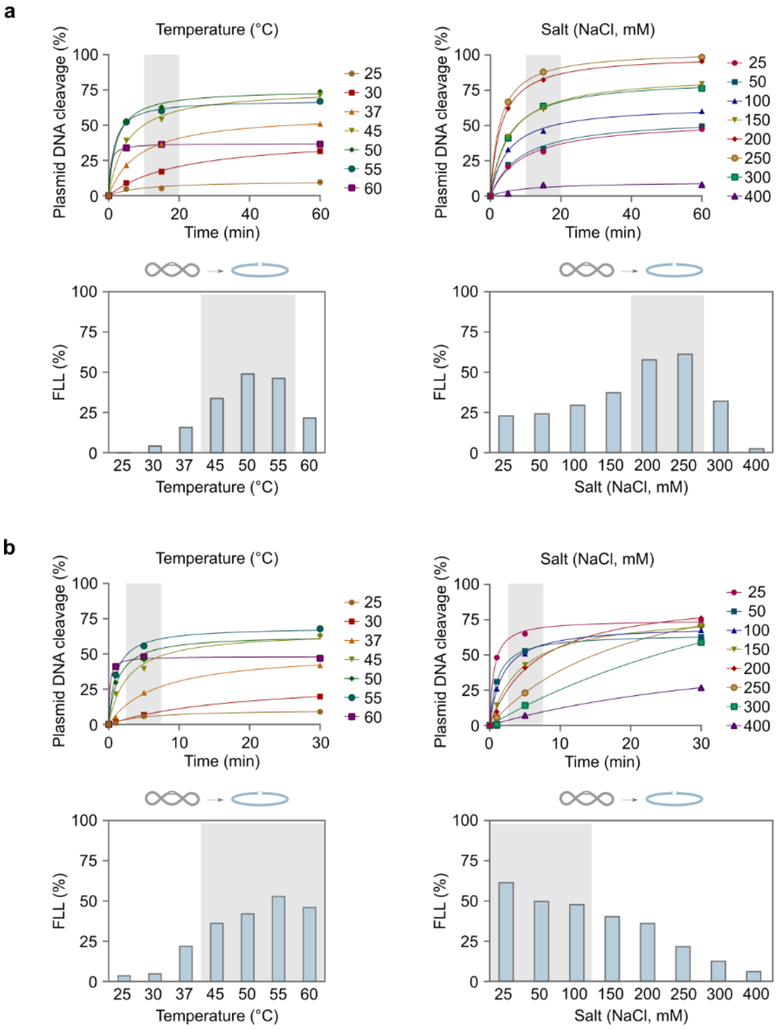

Supplementary Fig. 1 | SpCas12f1 and AsCas12f1 optimal plasmid DNA cleavage conditions in vitro. SpCas12f1 (**a**) and AsCas12f1 (**b**) RNP plasmid DNA cleavage was assayed by independently varying temperature (with 100 mM NaCl) and NaCl concentration (at 45°C temperature). In the line graphs, grey areas represent the time point used in the histograms to compare the efficiency of full-length linear (FLL) DNA cleavage under the different conditions. The grey areas in the histograms represent the optimal biochemical conditions for Cas12f1 cleavage activity. Cas12f1 RNP complexes were assembled using 20 nt spacer gRNAs.

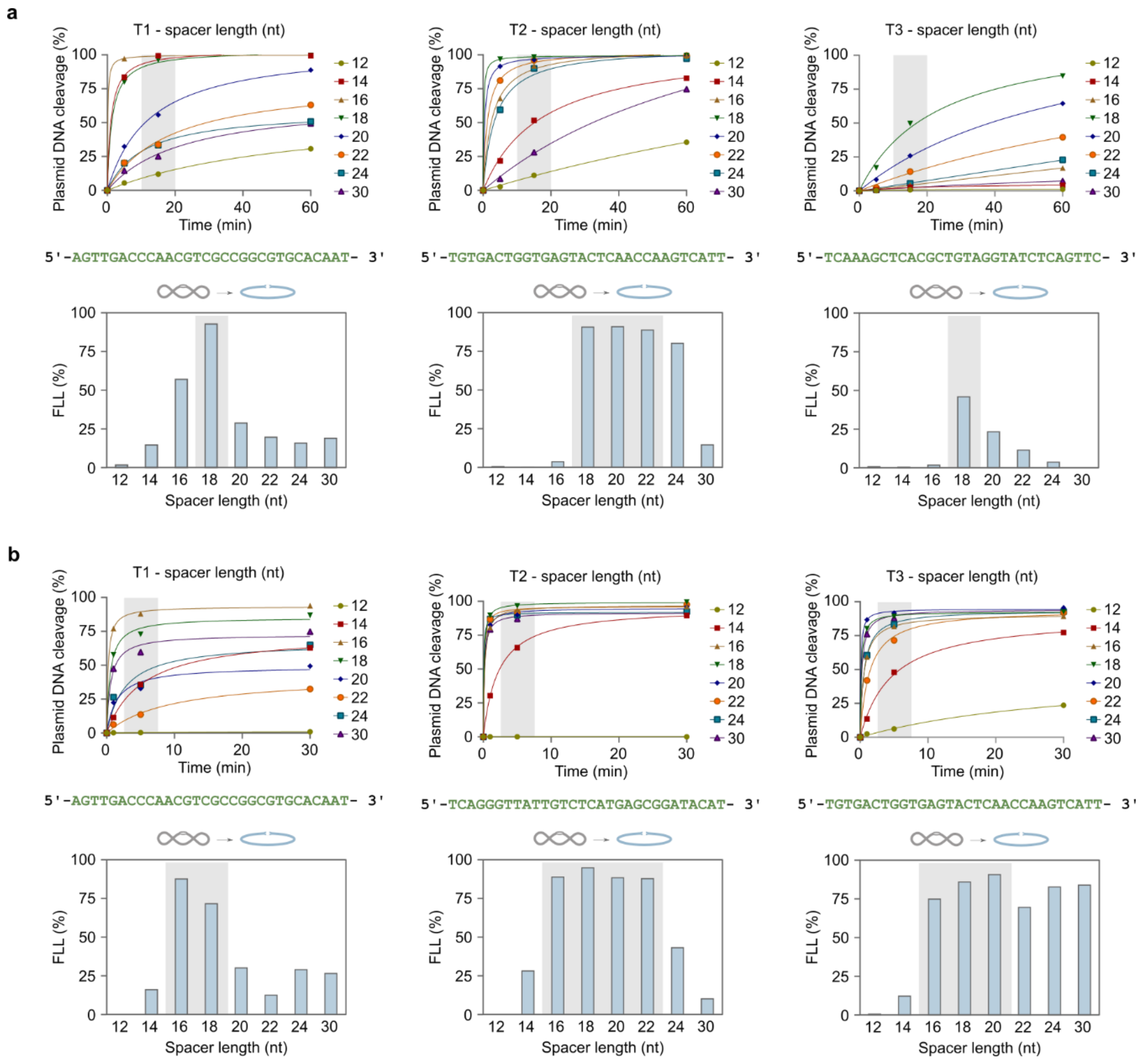

Supplementary Fig. 2 | Optimal Cas12f1 gRNA spacer length. SpCas12f1 (**a**) and AsCas12f1 (**b**) RNP complexes were assembled and used to assess dsDNA cleavage efficiency for different target sequences. Spacer length was varied from 12 to 30 nt in 2 nt increments for three different protospacer targets (shown in green). Marked grey areas in the line graphs represent the time point used for the corresponding histograms to assess the efficiency of full-length linear (FLL) DNA cleavage. The grey areas in the histograms show the optimal spacer length for each target.

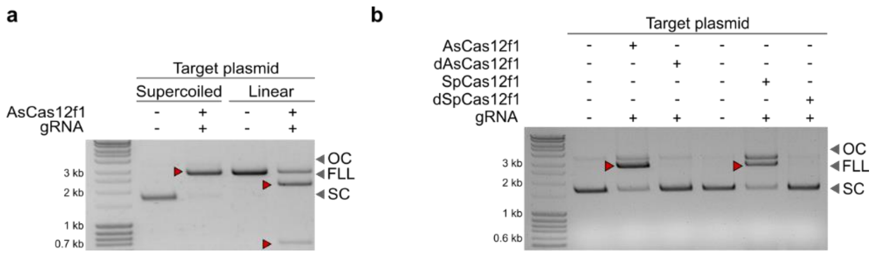

Supplementary Fig. 3 | Cas12f1 cleavage of double-stranded DNA targets. a, AsCas12f1 RNP complex efficiently cleaves supercoiled and linear plasmid dsDNA targets. **b**, Alanine substitution of conserved RuvC active site residues (dAsCas12f1 – D225A, dSpCas12f1 – D228A) completely abolishes DNA cleavage activity for both Cas12f1 nucleases. Cas12f1 RNP complexes were assembled using gRNAs with 18 nt spacers. SC, OC, and FLL stand for supercoiled, open-circle, and full-length linear, respectively. Source data are provided as a Source Data file.

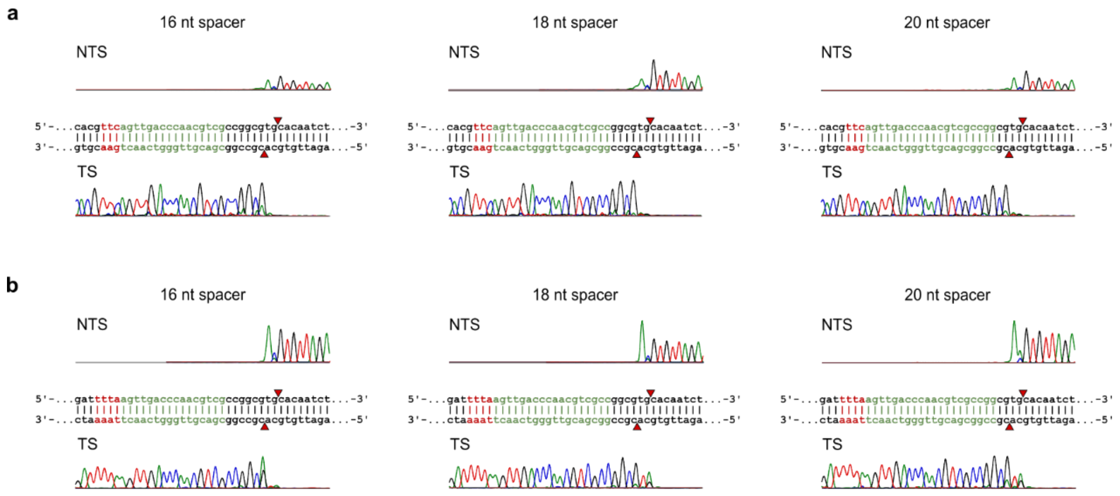

Supplementary Fig. 4 | Run-off sequencing of Cas12f1 cleaved plasmid DNA. Plasmid DNA cleavage with SpCas12f1 (**a**) and AsCas12f1 (**b**) RNP complexes resulted in a double-stranded break centered around positions 22-24 bp 3' from the PAM. The cleavage pattern was independent from gRNA spacer length for both Cas12f1 nucleases. NTS and TS represent non-target strand and target strand, respectively. The PAM is represented in red color, while the target sequence is shown in green.

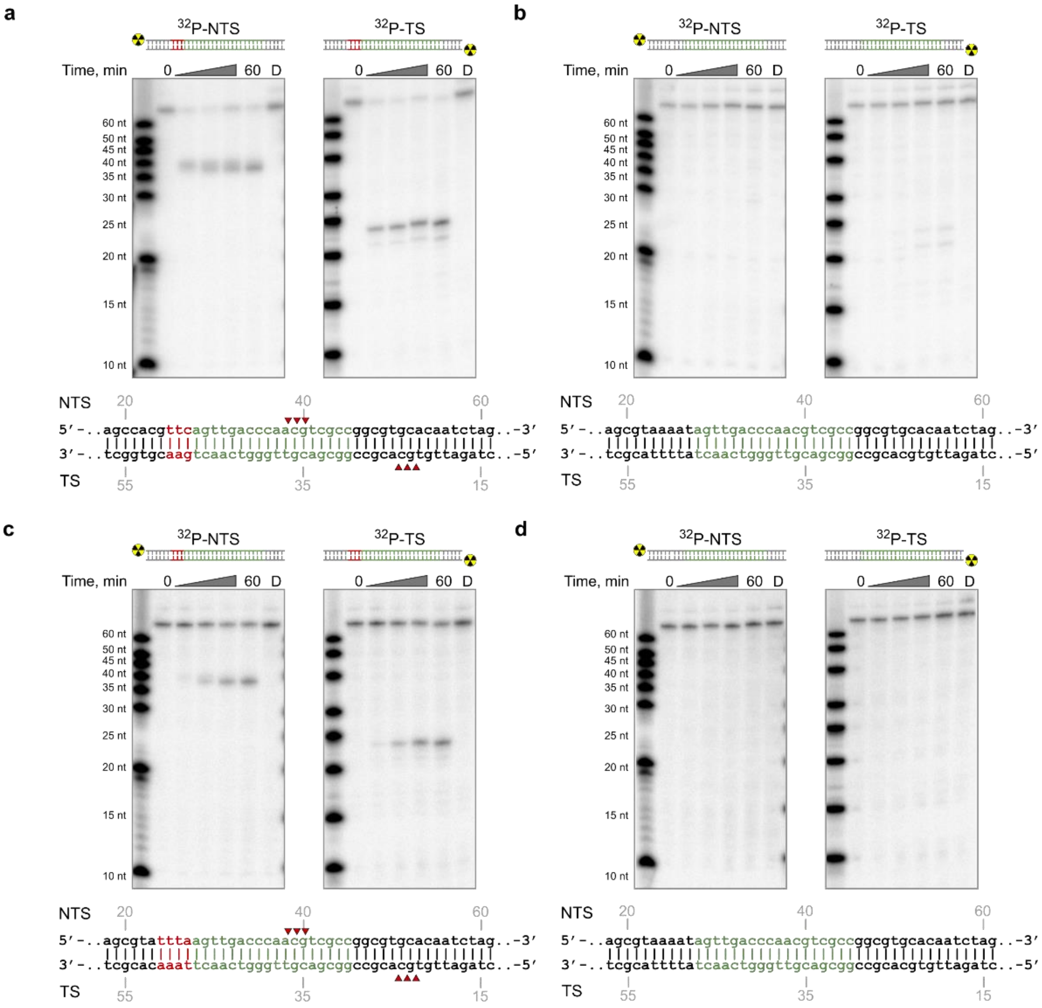

Supplementary Fig. 5 | Oligoduplex cleavage by Cas12f1 RNP complexes. Cleavage of radiolabeled dsDNA oligoduplexes by in vitro assembled SpCas12f1 (**a, b**) and AsCas12f1 (**c, d**) RNP complexes. Both Cas12f1 RNP complexes required target sequence (marked in green) and PAM (red) to efficiently cleave dsDNA. Cas12f1 RNP complexes were assembled using 18 nt spacer gRNAs. NTS and TS represent non-target strand and target strand, respectively, D – catalytically dead (d) Cas12f1 RNP complex (dSpCas12f1 – D228A, dAsCas12f1 – D225A), which was incubated with the DNA substrate for 60 min. Source data are provided as a Source Data file.

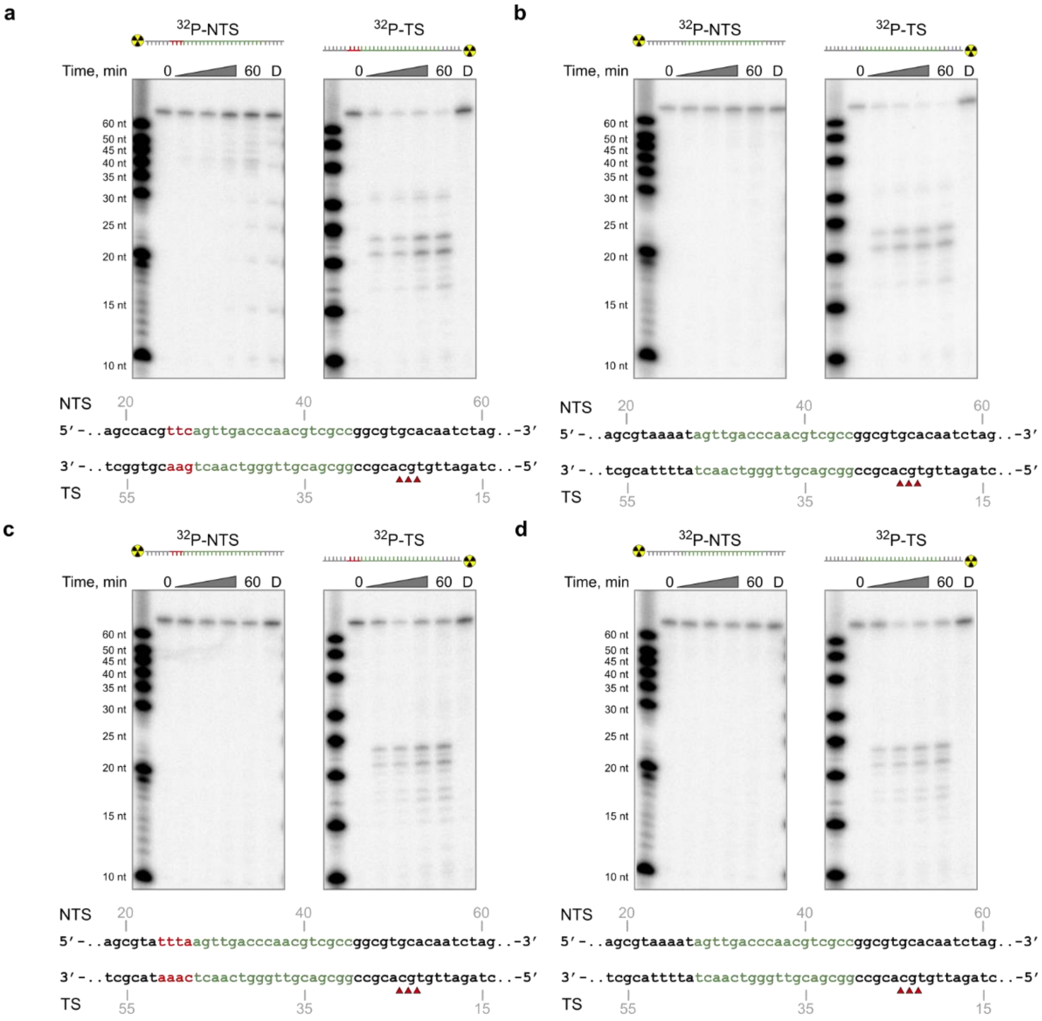

Supplementary Fig. 6 | ssDNA cleavage by Cas12f1 RNP complexes. Cleavage of radiolabeled ssDNA by in vitro assembled SpCas12f1 (**a, b**) and AsCas12f1 (**c, d**) RNP complexes. Substrates containing complementary to gRNA sequence (marked in green) were efficiently cleaved in a PAM (red) independent manner. Cas12f1 RNP complexes were assembled using 18 nt spacer gRNAs. NTS and TS represent non-target strand and target strand, respectively, D - dCas12f1 RNP complex (dSpCas12f1 – D228A, dAsCas12f1 – D225A), which was incubated with DNA substrate for 60 min. Source data are provided as a Source Data file.

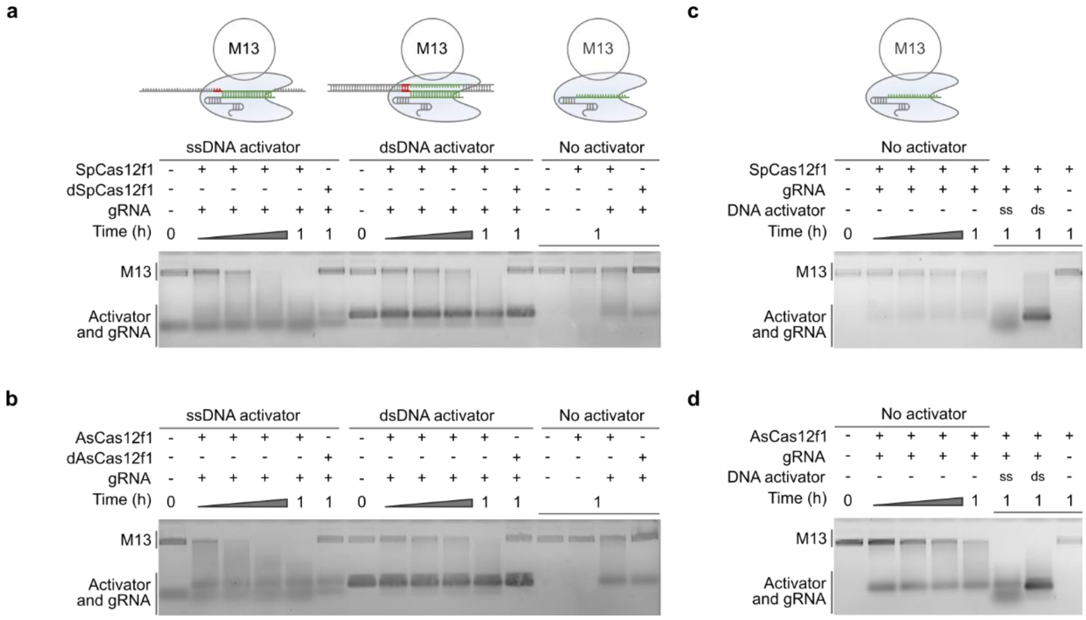

Supplementary Fig. 7 | Collateral ssDNA cleavage activity of Cas12f1 RNP complexes. Collateral non-specific M13 ssDNA degradation activity by SpCas12f1 (**a**) and AsCas12f1 (**b**) is triggered by ssDNA or PAM-containing dsDNA targets. As observed for Cas12a (Chen *et al*., *Science*, 360: 436-439, 2018), slight nuclease activity against non-specific ssDNA can be seen without any DNA activator for SpCas12f1 (**c**) and AsCas12f1 (**d**). dCas12f1 RNP complex (dSpCas12f1 – D228A and dAsCas12f1 – D225A) did not degrade ssDNA. Cas12f1 and dCas12f1 RNP complexes were assembled using gRNAs with 18 nt long spacers. Source data are provided as a Source Data file.

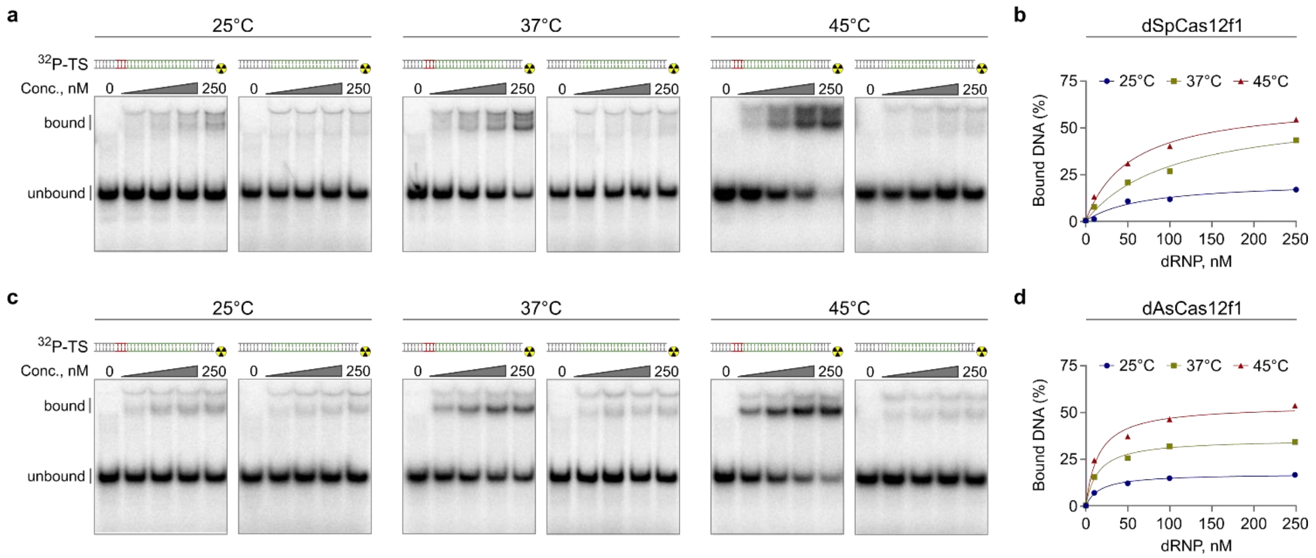

Supplementary Fig. 8 | Temperature dependent dsDNA binding by Cas12f1 RNP complexes. dsDNA binding experiment of dSpCas12f1 (D228A) (**a, b**) and dAsCas12f1 (D225A) (**c, d**) RNP complexes. Different amounts of dSpCas12f1 and dAsCas12f1 RNP complexes were pre-incubated with 1 nM of ^{32}P-5′-labeled dsDNA substrates at the indicated temperatures. Samples were analyzed by non-denaturing PAGE (polyacrylamide gel electrophoresis) at room temperature. Schematic representation of the DNA substrates is shown above the corresponding gel (PAM shown in red color, target in green). dCas12f1 RNP complexes were assembled using gRNAs with 18 nt long spacers. Source data are provided as a Source Data file.

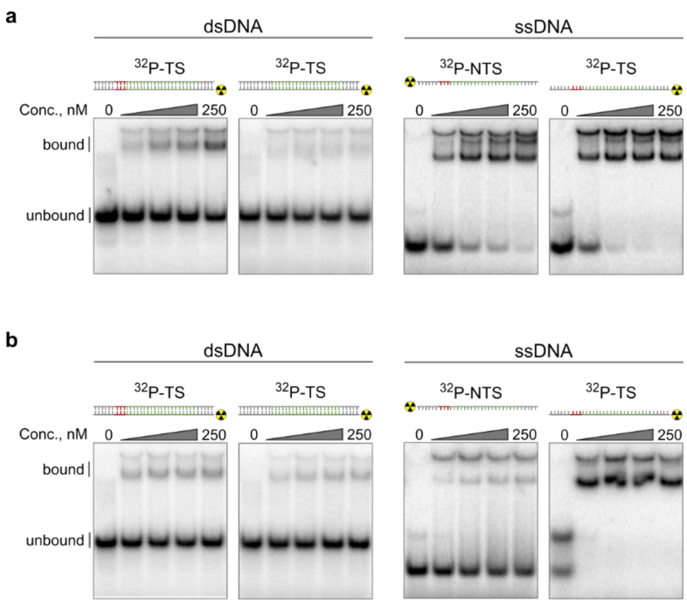

Supplementary Fig. 9 | ds and ssDNA binding activity of Cas12f1 RNP complexes. dsDNA and ssDNA binding by dSpCas12f1 (D228A) (**a**) and dAsCas12f1 (D225A) (**b**) RNP complexes. dSpCas12f1 and dAsCas12f1 both preferentially bind PAM containing dsDNA targets. For ssDNA, dAsCas12f1 binds more strongly to the TS (**b**), while dSpCas12f1 binds both TS and NTS with similar affinity (**a**). Different amounts of dSpCas12f1 and dAsCas12f1 RNP complexes were pre-incubated with 1 nM of ^{32}P-5′-labeled ds or ssDNA substrates at room temperature and analyzed by non-denaturing PAGE (polyacrylamide gel electrophoresis). DNA substrates are shown schematically above the corresponding gel (PAM shown in red color, target in green). NTS and TS represent non-target strand and target strand, respectively. dCas12f1 RNP complexes were assembled using gRNAs with 18 nt long spacers. Source data are provided as a Source Data file.

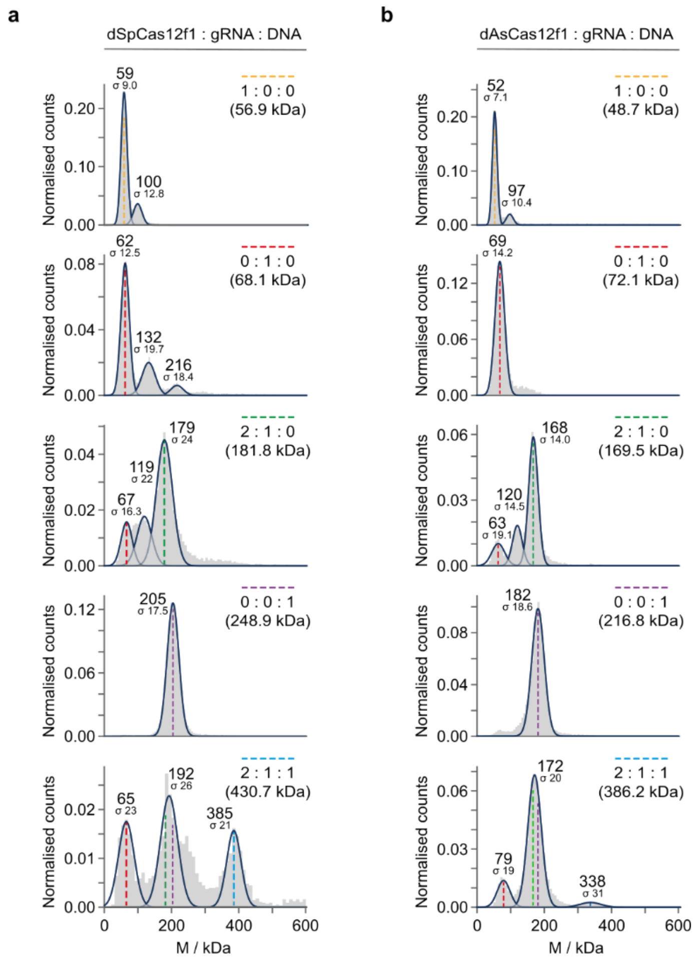

Supplementary Fig. 10 | Molecular weight measurements of Cas12f1 protein and RNP complexes using mass photometry. Molecular mass distributions obtained for dSpCas12f1 (D228A) (**a**) and dAsCas12f1 (D225A) (**b**). Colored dashed lines indicate the observed molecular weights for the different, components: yellow – dCas12f1, red – gRNA, green – dCas12f1-gRNA binary complex, purple – dsDNA containing specific PAM and target sequences, blue – dCas12f1-gRNA-DNA ternary complex. Theoretical masses of the main species are shown in brackets for the given stoichiometries. dCas12f1 RNP complexes were assembled using gRNAs with 18 nt long spacers.

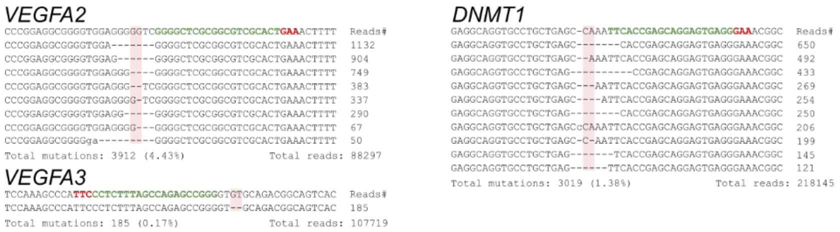

Supplementary Fig. 11 | SpCas12f1 genome editing in HEK293T cells. HEK293T cells were transfected with DNA expression construct containing SpCas12f1 and gRNA. Amplicon sequencing data yielded indels for three chosen target sites where PAM is marked in red and gRNA target sequence in green. The expected position of cleavage is shaded in red while lowercase nucleotides represent added DNA or insertions.

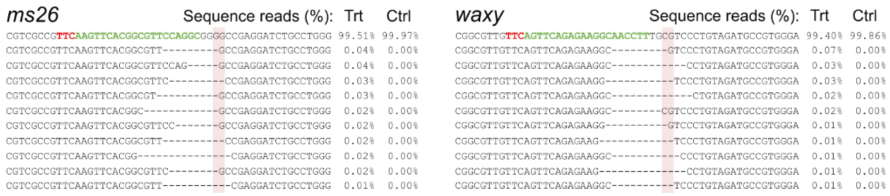

Supplementary Fig. 12 | SpCas12f1 transient editing in *Zea mays* cells. Alignment of sequence reads shows the presence of insertion of deletion (indel) mutations originating at or spanning the predicted cut-site for both *ms26* and *waxy* targets. The observed indel mutations from the 45°C SpCas12f1 treatments (Trt) weren't identified in control (Ctrl) experiments (where the expression cassette encoding the gRNA was omitted). PAM is shown in red and protospacer target in green. The expected position of cleavage is shaded in red.

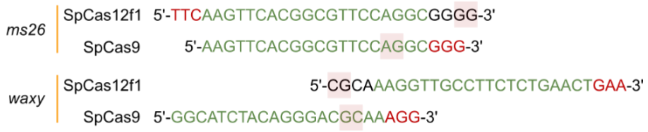

Supplementary Fig. 13 | Overlap of SpCas12f1 and SpCas9 *ms26* and *waxy* targets. The PAM is shown in red and the protospacer target in green. The expected position of cleavage is shaded in red.

Supplementary Table 1 | Plasmids used in this study.

Plasmid name	Description	Figures	Link
pMBP-SpCas12f1	SpCas12f1 expression	1B, 2, S1A, S2A, S3B, S4A, S5A, S6A, S7A&C	https://benchling.com/s/seq-zUwkN19bRbbTxepibRqf
pMBP-AsCas12f1	AsCas12f1 expression	1B, S1B, S2B, S3, S4B, S5B, S6A, S7B&D	https://benchling.com/s/seq-xGf89cD2PL7Xob1ir3M4
pGB-070	SpCas12f1 D228A expression	2D&E, S3B, S5A, S6A, S7A, S8A&C, S9A, S10A	https://benchling.com/s/seq-BILfejnI93meTV5EyZY0
pGB-069	AsCas12f1 D225A expression	S3B, S5B, S6B, S7B, S8B&D, S9B, S10B	https://benchling.com/s/seq-67HDmF2WxfEkTSvHk868
pSpCas12f1-pETduet-1	SpCas12f1 intact locus (T7 expression)	1B	https://benchling.com/s/seq-XHumnO4wkaHa2vxa7QKZ
pAsCas12f1-pETduet-1	AsCas12f1 intact locus (T7 expression)	1B	https://benchling.com/s/seq-2bIGj5WAhZLmAk8wHEhT
pKP14	Target plasmid for SpCas12f1	2A-C, S1A, S2A, S3B, S4A	https://benchling.com/s/seq-EPL613Wf6V5H0rhREGSL
pKP12	Target plasmid for AsCas12f1	S1B, S2B, S3A&B, S4B	https://benchling.com/s/seq-W6Xc8gCZerTaW3iBdzSr
pRZ-101-AsCas12a-NT	Plasmid for AsCas12a and non-targeting gRNA expression in human cells	3B	https://benchling.com/s/seq-yDONKunfOfNYLlbmY55V
pRZ-162-AsCas12a-VEGFA2	Plasmid for AsCas12a and VEGFA2 gRNA expression in human cells	3B	https://benchling.com/s/seq-Q5KMPMN4hSv08GwiIdF5
pRZ-102-AsCas12a-VEGFA3	Plasmid for AsCas12a and VEGFA3 gRNA expression in human cells	3B	https://benchling.com/s/seq-fIifXrX9hgNlqdA9JLZa
pRZ-104-AsCas12a-DNMT1	Plasmid for AsCas12a and DNMT1 gRNA expression in human cells	3B	https://benchling.com/s/seq-zhSFfZLra1QZa8UcPrqE
pRZ-105-AsCas12f1-NT	Plasmid for AsCas12f1 and non-targeting gRNA expression in human cells	3B	https://benchling.com/s/seq-YcM3Dnsxs2GipEASEcxO

pRZ-107-AsCas12f1-VEFGA2	Plasmid for AsCas12f1 and VEGFA2 gRNA expression in human cells	3B	https://benchling.com/s/seq-J9AcGWXwBKYiK2mdxCHp
pRZ-106-AsCas12f1-VEGFA3	Plasmid for AsCas12f1 and VEGFA3 gRNA expression in human cells	3B	https://benchling.com/s/seq-pI7JRUBc2XZHogVmgSBP
pRZ-108-AsCas12f1-DNMT1	Plasmid for AsCas12f1 and DNMT1 gRNA expression in human cells	3B	https://benchling.com/s/seq-TwRn8F0l3Al0jMdrv8ue
pRZ-109-SpCas12f1-NT	Plasmid for SpCas12f1 and non-targeting gRNA expression in human cells	3B	https://benchling.com/s/seq-BlFo4HyxYmDaTO5B5exo
pRZ-111-SpCas12f1-VEGFA2	Plasmid for SpCas12f1 and VEGFA2 gRNA expression in human cells	3B	https://benchling.com/s/seq-wT5Z40UW0e19mmw1i2MZ
pRZ-110-SpCas12f1-VEGFA3	Plasmid for SpCas12f1 and VEGFA3 gRNA expression in human cells	3B	https://benchling.com/s/seq-RMm93GUcIusNgBEsyKWD
pRZ-112-SpCas12f1-DNMT1	Plasmid for SpCas12f1 and DNMT1 gRNA expression in human cells	3B	https://benchling.com/s/seq-BZY0Dqhovc7uC7O1Alei
RV039055	Plasmid for SpCas12f1 protein expression in *Zea mays*	3D-G	https://benchling.com/s/seq-5x8vhcIhUFgBrRlFOAKX
RV008870_ms26	Plasmid for SpCas12f1 ms26 target gRNA expression in *Zea mays*	3D-G	https://benchling.com/s/seq-ubbCfOYchZrojU6ykpPG
RV008870_waxy	Plasmid for SpCas12f1 waxy target gRNA expression in *Zea mays*	3D-G	https://benchling.com/s/seq-50o7xJeDOiXNghsFFgBs
RV035712	Plasmid for SpCas9 protein expression in *Zea mays*	3E	https://benchling.com/s/seq-kFFZb6L5eJZRzquUxU37
RV008870-Cas9_ms26	Plasmid for SpCas9 ms26 target gRNA expression in *Zea mays*	3E	https://benchling.com/s/seq-6YJqLeddN4YZbvXojTHq
RV022942	Plasmid for SpCas9 waxy target gRNA expression in *Zea mays*	3E	https://benchling.com/s/seq-o5Rfl59cokCCOZnfXJ0l

Supplementary Table 2 | Sequences of the proteins used in this study.

SpCas12f1

MGESVKAIKLKILDMFLDPECTKQDDNWRKDLSTMSRFCAEAGNMCLRDLYNYFSMPKEDRISSKDLYNAMY
HKTKLLHPELPGKVANQIVNHAKDVWKRNAKLIYRNQISMPTYKITTAPIRLQNNIYKLIKNKNKYIIDVQLYSKE
YSKDSGKGTHRYFLVAVRDSSTRMIFDRIMSKDHIDSSKSYTQGQLQIKKDHQGKWYCIIPYTFPTHETVLDPD
KVMGVDLGVAKAVYWAFNSSYKRGCIDGGEIEHFRKMIRARRVSIQNQIKHSGDARKGHGRKRALKPIETLSE
KEKNFRDTINHRYANRIVEAAIKQGCGTIQIENLEGIADTTGSKFLKNWPYYDLQTKIVNKAKEHGITVVAINPQY
TSQRCSMCGYIEKTNRSSQAVFECKQCGYGSRTICINCRHVQVSGDVCEECGGIVKKENVNADYNAAKNIST
PYIDQIIMEKCLELGIPYRSITCKECGHIQASGNTCEVCGSTNILKPKKIRKAK*

10×His:MBP:TEV:SpCas12f1

MKSSHHHHHHHHHHGSSMKIEEGKLVIWINGDKGYNGLAEVGKKFEKDTGIKVTVEHPDKLEEKFPQVAATG
DGPDIIFWAHDRFGGYAQSGLLAEITPDKAFQDKLYPFTWDAVRYNGKLIAYPIAVEALSLIYNKDLLPNPPKT
WEEIPALDKELKAKGKSALMFNLQEPYFTWPLIAADGGYAFKYENGKYDIKDVGVDNAGAKAGLTFLVDLIKN
KHMNADTDYSIAEAAFNKGETAMTINGPWAWSNIDTSKVNYGVTVLPTFKGQPSKPFVGVLSAGINAASPNKE
LAKEFLENYLLTDEGLEAVNKDKPLGAVALKSYEEELAKDPRIAATMENAQKGEIMPNIPQMSAFWYAVRTAVI
NAASGRQTVDEALKDAQTNSSSNNNNNNNNNNLGIEENLYFQSNAMGESVKAIKLKILDMFLDPECTKQDDN
WRKDLSTMSRFCAEAGNMCLRDLYNYFSMPKEDRISSKDLYNAMYHKTKLLHPELPGKVANQIVNHAKDVW
KRNAKLIYRNQISMPTYKITTAPIRLQNNIYKLIKNKNKYIIDVQLYSKEYSKDSGKGTHRYFLVAVRDSSTRMIF
DRIMSKDHIDSSKSYTQGQLQIKKDHQGKWYCIIPYTFPTHETVLDPDKVMGVDLGVAKAVYWAFNSSYKRG
CIDGGEIEHFRKMIRARRVSIQNQIKHSGDARKGHGRKRALKPIETLSEKEKNFRDTINHRYANRIVEAAIKQGC
GTIQIENLEGIADTTGSKFLKNWPYYDLQTKIVNKAKEHGITVVAINPQYTSQRCSMCGYIEKTNRSSQAVFEC
KQCGYGSRTICINCRHVQVSGDVCEECGGIVKKENVNADYNAAKNISTPYIDQIIMEKCLELGIPYRSITCKECG
HIQASGNTCEVCGSTNILKPKKIRKAK*

AsCas12f1

MIKVYRYEIVKPLDLDWKEFGTILRQLQQETRFALNKATQLAWEWMGFSSDYKDNHGEYPKSKDILGYTNVH
GYAYHTIKTKAYRLNSGNLSQTIKRATDRFKAYQKEILRGDMSIPSYKRDIPLDLIKENISVNRMNHGDYIASLSL
LSNPAKQEMNVKRKISVIIIVRGAGKTIMDRILSGEYQVSASQIIHDDRKNKWYLNISYDFEPQTRVLDLNKIMGI
DLGVAVAVYMAFQHTPARYKLEGGEIENFRRQVESRRISMLRQGKYAGGARGGHGRDKRIKPIEQLRDKIAN
FRDTTNHRYSRYIVDMAIKEGCGTIQMEDLTNIRDIGSRFLQNWTYYDLQQKIIYKAEEAGIKVIKIDPQYTSQRC
SECGNIDSGNRIGQAIFKCRACGYEANADYNAARNIAIPNIDKIIAESIK*

10×His:MBP:TEV:AsCas12f1

MKSSHHHHHHHHHHGSSMKIEEGKLVIWINGDKGYNGLAEVGKKFEKDTGIKVTVEHPDKLEEKFPQVAATG
DGPDIIFWAHDRFGGYAQSGLLAEITPDKAFQDKLYPFTWDAVRYNGKLIAYPIAVEALSLIYNKDLLPNPPKT
WEEIPALDKELKAKGKSALMFNLQEPYFTWPLIAADGGYAFKYENGKYDIKDVGVDNAGAKAGLTFLVDLIKN
KHMNADTDYSIAEAAFNKGETAMTINGPWAWSNIDTSKVNYGVTVLPTFKGQPSKPFVGVLSAGINAASPNKE
LAKEFLENYLLTDEGLEAVNKDKPLGAVALKSYEEELAKDPRIAATMENAQKGEIMPNIPQMSAFWYAVRTAVI
NAASGRQTVDEALKDAQTNSSSNNNNNNNNNNLGIEENLYFQSNAMIKVYRYEIVKPLDLDWKEFGTILRQLQ
QETRFALNKATQLAWEWMGFSSDYKDNHGEYPKSKDILGYTNVHGYAYHTIKTKAYRLNSGNLSQTIKRATD
RFKAYQKEILRGDMSIPSYKRDIPLDLIKENISVNRMNHGDYIASLSLLSNPAKQEMNVKRKISVIIIVRGAGKTIM
DRILSGEYQVSASQIIHDDRKNKWYLNISYDFEPQTRVLDLNKIMGIDLGVAVAVYMAFQHTPARYKLEGGEIE
NFRRQVESRRISMLRQGKYAGGARGGHGRDKRIKPIEQLRDKIANFRDTTNHRYSRYIVDMAIKEGCGTIQME
DLTNIRDIGSRFLQNWTYYDLQQKIIYKAEEAGIKVIKIDPQYTSQRCSECGNIDSGNRIGQAIFKCRACGYEAN
ADYNAARNIAIPNIDKIIAESIK*

Supplementary Table 3 | RNAs used in this study.

Target	Spacer, nt	Sequence 5’-3’ (Target)	Figures
		**SpCas12f1**	
T1	12	GGGAUUUACUCUGUUUCGCGCGCCAGGGCAGUUAGGUGCCC<br>UAAAAGAGCGAAGUGGCCGAAAGGAAAGGCUAACGCUUCUCU<br>AACGCUACGGCGACCUUGGCGAAAUGCCAUCAAUACCACGC<br>GGCCCGAAAGGGUUCGCGCGAAACUGAGUAAUGAAAGUCGC<br>AUCUUGCGUAAGCGCGUGGAUUGAAACAGUUGACCCAAC	2A, S2A
	14	GGGAUUUACUCUGUUUCGCGCGCCAGGGCAGUUAGGUGCCC<br>UAAAAGAGCGAAGUGGCCGAAAGGAAAGGCUAACGCUUCUCU<br>AACGCUACGGCGACCUUGGCGAAAUGCCAUCAAUACCACGC<br>GGCCCGAAAGGGUUCGCGCGAAACUGAGUAAUGAAAGUCGC<br>AUCUUGCGUAAGCGCGUGGAUUGAAACAGUUGACCCAACGU	2A, S2A
	16	GGGAUUUACUCUGUUUCGCGCGCCAGGGCAGUUAGGUGCCC<br>UAAAAGAGCGAAGUGGCCGAAAGGAAAGGCUAACGCUUCUCU<br>AACGCUACGGCGACCUUGGCGAAAUGCCAUCAAUACCACGC<br>GGCCCGAAAGGGUUCGCGCGAAACUGAGUAAUGAAAGUCGC<br>AUCUUGCGUAAGCGCGUGGAUUGAAACAGUUGACCCAACGU<br>CG	2A, S2A, S4A
	18	GGGAUUUACUCUGUUUCGCGCGCCAGGGCAGUUAGGUGCCC<br>UAAAAGAGCGAAGUGGCCGAAAGGAAAGGCUAACGCUUCUCU<br>AACGCUACGGCGACCUUGGCGAAAUGCCAUCAAUACCACGC<br>GGCCCGAAAGGGUUCGCGCGAAACUGAGUAAUGAAAGUCGC<br>AUCUUGCGUAAGCGCGUGGAUUGAAACAGUUGACCCAACGU<br>CGCC	2, S1A, S2A, S3B, S4A, S5A, S6A, S7A&C, S8A&C, S9A, S10A
	20	GGGAUUUACUCUGUUUCGCGCGCCAGGGCAGUUAGGUGCCC<br>UAAAAGAGCGAAGUGGCCGAAAGGAAAGGCUAACGCUUCUCU<br>AACGCUACGGCGACCUUGGCGAAAUGCCAUCAAUACCACGC<br>GGCCCGAAAGGGUUCGCGCGAAACUGAGUAAUGAAAGUCGC<br>AUCUUGCGUAAGCGCGUGGAUUGAAACAGUUGACCCAACGU<br>CGCCGG	2A, S2A, S4A
	22	GGGAUUUACUCUGUUUCGCGCGCCAGGGCAGUUAGGUGCCC<br>UAAAAGAGCGAAGUGGCCGAAAGGAAAGGCUAACGCUUCUCU<br>AACGCUACGGCGACCUUGGCGAAAUGCCAUCAAUACCACGC<br>GGCCCGAAAGGGUUCGCGCGAAACUGAGUAAUGAAAGUCGC<br>AUCUUGCGUAAGCGCGUGGAUUGAAACAGUUGACCCAACGU<br>CGCCGGCG	2A, S2A
	24	GGGAUUUACUCUGUUUCGCGCGCCAGGGCAGUUAGGUGCCC<br>UAAAAGAGCGAAGUGGCCGAAAGGAAAGGCUAACGCUUCUCU<br>AACGCUACGGCGACCUUGGCGAAAUGCCAUCAAUACCACGC<br>GGCCCGAAAGGGUUCGCGCGAAACUGAGUAAUGAAAGUCGC	2A, S2A

		AUCUUGCGUAAGCGCGUGGAUUGAAACAGUUGACCCAACGUCGCCGGCGUG	
	30	GGGAUUUACUCUGUUUCGCGCGCCAGGGCAGUUAGGUGCCCUAAAAGAGCGAAGUGGCCGAAAGGAAAGGCUAACGCUUCUCUAACGCUACGGCGACCUUGGCGAAAUGCCAUCAAUACCACGCGGCCCGAAAGGGUUCGCGCGAAACUGAGUAAUGAAAGUCGCAUCUUGCGUAAGCGCGUGGAUUGAAACAGUUGACCCAACGUCGCCGGCGUGCACAAU	2A, S2A
T2	12, 14, 16, 18, 20, 22, 24, 30	GGGAUUUACUCUGUUUCGCGCGCCAGGGCAGUUAGGUGCCCUAAAAGAGCGAAGUGGCCGAAAGGAAAGGCUAACGCUUCUCUAACGCUACGGCGACCUUGGCGAAAUGCCAUCAAUACCACGCGGCCCGAAAGGGUUCGCGCGAAACUGAGUAAUGAAAGUCGCAUCUUGCGUAAGCGCGUGGAUUGAAACUGUGACUGGUGAGUACUCAACCAAGUCAUU	S2A
T3	12, 14, 16, 18, 20, 22, 24, 30	GGGAUUUACUCUGUUUCGCGCGCCAGGGCAGUUAGGUGCCCUAAAAGAGCGAAGUGGCCGAAAGGAAAGGCUAACGCUUCUCUAACGCUACGGCGACCUUGGCGAAAUGCCAUCAAUACCACGCGGCCCGAAAGGGUUCGCGCGAAACUGAGUAAUGAAAGUCGCAUCUUGCGUAAGCGCGUGGAUUGAAACUCAAAGCUCACGCUGUAGGUAUCUCAGUUC	S2A
**AsCas12f1**			
T1	12	GGGAUACUUCUAUUCGUCGGUUCAGCGACGAUAAGCCGAGAAGUGCCAAUAAAACUGUUAAGUGGUUUGGUAACGCUCGGUAAGGUAGCCAAAAGGCUGAAACUCCGUGCACAAAGACCGCACGGACGCUUCACAUAUAGCUCAUAAACAAAUGUCGUCGACCUCUAAUAGCGAAAGUUUGCGAGCUAGCUUGUGGAGUGUGAACAGUUGACCCAAC	S2B
	14	GGGAUACUUCUAUUCGUCGGUUCAGCGACGAUAAGCCGAGAAGUGCCAAUAAAACUGUUAAGUGGUUUGGUAACGCUCGGUAAGGUAGCCAAAAGGCUGAAACUCCGUGCACAAAGACCGCACGGACGCUUCACAUAUAGCUCAUAAACAAAUGUCGUCGACCUCUAAUAGCGAAAGUUUGCGAGCUAGCUUGUGGAGUGUGAACAGUUGACCCAACGU	S2B
	16	GGGAUACUUCUAUUCGUCGGUUCAGCGACGAUAAGCCGAGAAGUGCCAAUAAAACUGUUAAGUGGUUUGGUAACGCUCGGUAAGGUAGCCAAAAGGCUGAAACUCCGUGCACAAAGACCGCACGGACGCUUCACAUAUAGCUCAUAAACAAAUGUCGUCGACCUCUAAUAGCGAAAGUUUGCGAGCUAGCUUGUGGAGUGUGAACAGUUGACCCAACGUCG	S2B, S4B
	18	GGGAUACUUCUAUUCGUCGGUUCAGCGACGAUAAGCCGAGAAGUGCCAAUAAAACUGUUAAGUGGUUUGGUAACGCUCGGUAAGGUAGCCAAAAGGCUGAAACUCCGUGCACAAAGACCGCACGGACGCUUCACAUAUAGCUCAUAAACAAAUGUCGUCGACCUCUAAUAGCGAAAGUUUGCGAGCUAGCUUGUGGAGUGUGAACAGUUGACCCAACGUCGCC	S1B, S2B, S3, S4B, S5B, S6B, S7B&D, S8B&D, S9B, S10B

	20	GGGAUACUUCUAUUCGUCGGUUCAGCGACGAUAAGCCGAGA<br>AGUGCCAAUAAAACUGUUAAGUGGUUUGGUAACGCUCGGUA<br>AGGUAGCCAAAAGGCUGAAACUCCGUGCACAAAGACCGCACG<br>GACGCUUCACAUAUAGCUCAUAAACAAAUGUCGUCGACCUCU<br>AAUAGCGAAAGUUUGCGAGCUAGCUUGUGGAGUGUGAACAG<br>UUGACCCAACGUCGCCGG	S2B, S4B
	22	GGGAUACUUCUAUUCGUCGGUUCAGCGACGAUAAGCCGAGA<br>AGUGCCAAUAAAACUGUUAAGUGGUUUGGUAACGCUCGGUA<br>AGGUAGCCAAAAGGCUGAAACUCCGUGCACAAAGACCGCACG<br>GACGCUUCACAUAUAGCUCAUAAACAAAUGUCGUCGACCUCU<br>AAUAGCGAAAGUUUGCGAGCUAGCUUGUGGAGUGUGAACAG<br>UUGACCCAACGUCGCCGGCG	S2B
	24	GGGAUACUUCUAUUCGUCGGUUCAGCGACGAUAAGCCGAGA<br>AGUGCCAAUAAAACUGUUAAGUGGUUUGGUAACGCUCGGUA<br>AGGUAGCCAAAAGGCUGAAACUCCGUGCACAAAGACCGCACG<br>GACGCUUCACAUAUAGCUCAUAAACAAAUGUCGUCGACCUCU<br>AAUAGCGAAAGUUUGCGAGCUAGCUUGUGGAGUGUGAACAG<br>UUGACCCAACGUCGCCGGCGUG	S2B
	30	GGGAUACUUCUAUUCGUCGGUUCAGCGACGAUAAGCCGAGA<br>AGUGCCAAUAAAACUGUUAAGUGGUUUGGUAACGCUCGGUA<br>AGGUAGCCAAAAGGCUGAAACUCCGUGCACAAAGACCGCACG<br>GACGCUUCACAUAUAGCUCAUAAACAAAUGUCGUCGACCUCU<br>AAUAGCGAAAGUUUGCGAGCUAGCUUGUGGAGUGUGAACAG<br>UUGACCCAACGUCGCCGGCGUGCACAAU	S2B
T2	12, 14, 16, 18, 20, 22, 24, 30	GGGAUACUUCUAUUCGUCGGUUCAGCGACGAUAAGCCGAGA<br>AGUGCCAAUAAAACUGUUAAGUGGUUUGGUAACGCUCGGUA<br>AGGUAGCCAAAAGGCUGAAACUCCGUGCACAAAGACCGCACG<br>GACGCUUCACAUAUAGCUCAUAAACAAAUGUCGUCGACCUCU<br>AAUAGCGAAAGUUUGCGAGCUAGCUUGUGGAGUGUGAACUC<br>AGGGUUAUUGUCUCAUGAGCGGAUACAU	S2B
T3	12, 14, 16, 18, 20, 22, 24, 30	GGGAUACUUCUAUUCGUCGGUUCAGCGACGAUAAGCCGAGA<br>AGUGCCAAUAAAACUGUUAAGUGGUUUGGUAACGCUCGGUA<br>AGGUAGCCAAAAGGCUGAAACUCCGUGCACAAAGACCGCACG<br>GACGCUUCACAUAUAGCUCAUAAACAAAUGUCGUCGACCUCU<br>AAUAGCGAAAGUUUGCGAGCUAGCUUGUGGAGUGUGAACUG<br>CUUCCGGCUCGUAUGUUGUGUGGAAUUG	S2B

Supplementary Table 4 | Oligonucleotides and DNA activators used for different cleavage assays in this study.

Description	Sequence 5'-3' (PAM, Target)	Figures
Target (forward) for SpCas12f1	GCACCTTACTGCAAGGTAGCCACGTTCAGTTGACCCAACGTCGCCGGCGTGCACAATCTAGATGCATCAGCTGC	S5A, S6A, S8A&C, S9A
Target (reverse) for SpCas12f1	GCAGCTGATGCATCTAGATTGTGCACGCCGGCGACGTTGGGTCAACTGAACGTGGCTACCTTGCAGTAAGGTGC	S5A, S6A, S8A&C, S9A
Target (forward) for AsCas12f1	GCACCTTACTGCAAGGTAGCGTATTTAAGTTGACCCAACGTCGCCGGCGTGCACAATCTAGATGCATCAGCTGC	S5B, S6B, S8B&D, S9B
Target (reverse) for AsCas12f1	GCAGCTGATGCATCTAGATTGTGCACGCCGGCGACGTTGGGTCAACTTAAATACGCTACCTTGCAGTAAGGTGC	S5B, S6B, S8B&D, S9B
Control target (forward)	GCACCTTACTGCAAGGTAGCGTAAAATAGTTGACCCAACGTCGCCGGCGTGCACAATCTAGATGCATCAGCTGC	S5, S6, S9
Control target (reverse)	GCAGCTGATGCATCTAGATTGTGCACGCCGGCGACGTTGGGTCAACTATTTTACGCTACCTTGCAGTAAGGTGC	S5, S6, S9
Forward strand marker	GCACCTTACTGCAAGGTAGCGTATTTAAGTTGACCCAACGTCGCCGGCGTGCACAATCTA	S5, S6
	GCACCTTACTGCAAGGTAGCGTATTTAAGTTGACCCAACGTCGCCGGCGT	
	GCACCTTACTGCAAGGTAGCGTATTTAAGTTGACCCAACGTCGCC	
	GCACCTTACTGCAAGGTAGCGTATTTAAGTTGACCCAACG	
	GCACCTTACTGCAAGGTAGCGTATTTAAGTTGACC	
	GCACCTTACTGCAAGGTAGCGTATTTAAGT	
	GCACCTTACTGCAAGGTAGC	
	GCACCTTACT	
Reverse strand marker	GCAGCTGATGCATCTAGATTGTGCACGCCGGCGACGTTGGGTCAACTTAAATACGCTACC	S5, S6
	GCAGCTGATGCATCTAGATTGTGCACGCCGGCGACGTTGGGTCAACTTAA	
	GCAGCTGATGCATCTAGATTGTGCACGCCGGCGACGTTGG	
	GCAGCTGATGCATCTAGATTGTGCACGCCG	
	GCAGCTGATGCATCTAGATTGTGCA	
	GCAGCTGATGCATCTAGATT	

	GCAGCTGATGCATCT	
	GCAGCTGATG	
ssDNA activator for SpCas12f1	GCAGCTGATGCATCTAGATTGTGCACGCCGGCGACGTTGGGTCAACTGAACGTGGCTACCTTGCAGTAAGGTGC	S7A&C
dsDNA activator for SpCas12f1	GCCAGGGTTTTCCCAGTCACGACGTTGTAAAACGACGGCCAGTGCCAAGCTTGCATGCCTGCAGGTCAATTCCACGTTCAGTTGACCCAACGTCGCCGGCGTGCACAATCTAGATGCTAGGACTCTAGAGGATCCCCGGGTACCGAGCTCGAATTCGTAATCATGGTCATAGCTGTTTCCTGTGTGAAATTGTTATCCGCTCACAATTCCACACAACATACGAGCCGGAAGCATAAAGTGTAAAGCCTGGGGTGCCTAATGAGTGAGCTAACTCACATTAATTGCGTTGCGCTCACTGCCCGCTTTCCAGTCGGGAAACCTGTCGTGCCAGCTGCATTAATGAATCGGCCAACGCGCGGGGAGAGGCGGTTTGCGTATTGGGCGCTCTTCCGCTTCCTCGC	S7A&C
ssDNA activator for AsCas12f1	GCAGCTGATGCATCTAGATTGTGCACGCCGGCGACGTTGGGTCAACTTAAATACGCTACCTTGCAGTAAGGTGC	S7B&D
dsDNA activator for AsCas12f1	GCCAGGGTTTTCCCAGTCACGACGTTGTAAAACGACGGCCAGTGCCAAGCTTGCATGCCTGCAGGTCAATTCGATTTTAAGTTGACCCAACGTCGCCGGCGTGCACAATCTAGATGCTAGGACTCTAGAGGATCCCCGGGTACCGAGCTCGAATTCGTAATCATGGTCATAGCTGTTTCCTGTGTGAAATTGTTATCCGCTCACAATTCCACACAACATACGAGCCGGAAGCATAAAGTGTAAAGCCTGGGGTGCCTAATGAGTGAGCTAACTCACATTAATTGCGTTGCGCTCACTGCCCGCTTTCCAGTCGGGAAACCTGTCGTGCCAGCTGCATTAATGAATCGGCCAACGCGCGGGGAGAGGCGGTTTGCGTATTGGGCGCTCTTCCGCTTCCTCGC	S7B&D
dsDNA used for SpCas12f1 mass photometry assay	AAGGAGAAAATACCGCATCAGGCGCCATTCGCCATTCAGGCTGCGCAACTGTTGGGAAGGGCGATCGGTGCGGGCCTCTTCGCTATTACGCCAGCTGGCGAAAGGGGGATGTGCTGCAAGGCGATTAAGTTGGGTAACGCCAGGGTTTTCCCAGTCACGACGTTGTAAAACGACGGCCAGTGCCAAGCTTGCATGCCTGCAGGTCAATTCCACGTTCAGTTGACCCAACGTCGCCGGCGTGCACAATCTAGATGCTAGGACTCTAGAGGATCCCCGGGTACCGAGCTCGAATTCGTAATCATGGTCATAGCTGTTTCCTGTGTGAAATTGTTATCCGCTCACAATTCCACACAACATACGAGCCGGAAGCATAAAGTGTAAAGCCTGGGGTGCCTAATGAGTG	2E, S10A
dsDNA used for AsCas12f1mass photometry assay	AAGGAGAAAATACCGCATCAGGCGCCATTCGCCATTCAGGCTGCGCAACTGTTGGGAAGGGCGATCGGTGCGGGCCTCTTCGCTATTACGCCAGCTGGCGAAAGGGGGATGTGCTGCAAGGCGATTAAGTTGGGTAACGCCAGGGTTTTCCCAGTCACGACGTTGTAAAACGACGGCCAGTGCCAAGCTAGTATTTTAAGTTGACCCAACGTCGCCGGCGTGCACAATCTAGATGCATAATTCGTAATCATGGTCATAGCTGTTTCCTGTGTGAAATTGTTATCCGCTCACAATTCCACACAACATACGAGCCGGAAGCATAAAGTGTAAAGCCTGGGGTGCCTAATGAGTG	S10B

Supplementary Table 5 | Primers used in assessing human and maize cell editing. Blue font represents the sequence required for Illumina sequencing, green font shows the additional sequence included to ensure balanced read composition in initial rounds of Illumina sequencing and black font indicates the sequence responsible for annealing to the genomic locus.

**Description**	**Designation within set**	**Sequence 5'-3'**
Primary PCR VEGFA3 (SpCas12f1, AsCas12f1, AsCas12a)	NGS-F1	ATCGGGAAGCTGAAGATCTGCGTTTGGGAGGTCAGAAATA
	NGS-F2	ATCGGGAAGCTGAAGGCTCTAGTTTGGGAGGTCAGAAATA
	NGS-F3	ATCGGGAAGCTGAAGCGAGATGTTTGGGAGGTCAGAAATA
	NGS-F4	ATCGGGAAGCTGAAGTAGACGGTTTGGGAGGTCAGAAATA
	NGS-R	ATCCGACGGTAGTGTGACGTCCTCACTCTCGAAG
Primary PCR DNMT1 (SpCas12f1)	NGS-F1	ATCGGGAAGCTGAAGATCTGCCTCAAGTGAGCAGCTGAG
	NGS-F2	ATCGGGAAGCTGAAGGCTCTACTCAAGTGAGCAGCTGAG
	NGS-F3	ATCGGGAAGCTGAAGCGAGATCTCAAGTGAGCAGCTGAG
	NGS-F4	ATCGGGAAGCTGAAGTAGACGNCTCAAGTGAGCAGCTGAG
	NGS-R	ATCCGACGGTAGTGTGGTGAGGATTGAGTGAGTT
Primary PCR DNMT1 (AsCas12f1, AsCas12a)	NGS-F1	ATCGGGAAGCTGAAGATCTGCTGAACACTCCTCAAACGGTC
	NGS-F2	ATCGGGAAGCTGAAGGCTCTATGAACACTCCTCAAACGGTC
	NGS-F3	ATCGGGAAGCTGAAGCGAGATTGAACACTCCTCAAACGGTC
	NGS-F4	ATCGGGAAGCTGAAGTAGACGTGAACACTCCTCAAACGGTC
	NGS-R	ATCCGACGGTAGTGTGCCCTCACTCCTGCTCGGT
Primary PCR AGBL1 (SpCas12f1)	NGS-F1	ATCGGGAAGCTGAAGATCTGCGTTGACAATAACACAACAGG
	NGS-F2	ATCGGGAAGCTGAAGGCTCTAGTTGACAATAACACAACAGG
	NGS-F3	ATCGGGAAGCTGAAGCGAGATGTTGACAATAACACAACAGG
	NGS-F4	ATCGGGAAGCTGAAGTAGACGGTTGACAATAACACAACAGG
	NGS-R	ATCCGACGGTAGTGTGCCTTAAGGAAACAGAAGAG
Primary PCR VEGFA2 (SpCas12f1)	NGS-F1	ATCGGGAAGCTGAAGATCTGCAGCTACCACCTCCTCCCCGGC
	NGS-F2	ATCGGGAAGCTGAAGGCTCTAAGCTACCACCTCCTCCCCGGC
	NGS-F3	ATCGGGAAGCTGAAGCGAGATAGCTACCACCTCCTCCCCGGC
	NGS-F4	ATCGGGAAGCTGAAGTAGACGAGCTACCACCTCCTCCCCGGC
	NGS-R	ATCCGACGGTAGTGTGCGGCTCCTCCGAAGCGAGAAC

Primary PCR VEGFA2 (AsCas12f1 and AsCas12a)	NGS-F1	ATCGGGAAGCTGAAGATCTGCGGGCGTGCGAGCAGCGAAAG
	NGS-F2	ATCGGGAAGCTGAAGGCTCTAGGGCGTGCGAGCAGCGAAAG
	NGS-F3	ATCGGGAAGCTGAAGCGAGATGGGCGTGCGAGCAGCGAAAG
	NGS-F4	ATCGGGAAGCTGAAGTAGACGGGGCGTGCGAGCAGCGAAAG
	NGS-R	ATCCGACGGTAGTGTGTGTCCGTCAGCGCGACTGGT
Primary PCR FANCF (SpCas12f1)	NGS-F1	ATCGGGAAGCTGAAGATCTGCGCTACCTGCGCCACATCCA
	NGS-F2	ATCGGGAAGCTGAAGGCTCTAGCTACCTGCGCCACATCCA
	NGS-F3	ATCGGGAAGCTGAAGCGAGATGCTACCTGCGCCACATCCA
	NGS-F4	ATCGGGAAGCTGAAGTAGACGGCTACCTGCGCCACATCCA
	NGS-R	ATCCGACGGTAGTGTGACCGAGGGCCTGGAAGTTC
Primary PCR EMX1 (SpCas12f1)	NGS-F1	ATCGGGAAGCTGAAGATCTGCGCTCAGCCTGAGTGTTGAGGC
	NGS-F2	ATCGGGAAGCTGAAGGCTCTAGCTCAGCCTGAGTGTTGAGGC
	NGS-F3	ATCGGGAAGCTGAAGCGAGATGCTCAGCCTGAGTGTTGAGGC
	NGS-F4	ATCGGGAAGCTGAAGTAGACGGCTCAGCCTGAGTGTTGAGGC
	NGS-R	ATCCGACGGTAGTGTGTGGCCTGCTTCGTGGCAAT
Primary PCR ms26 (SpCas12f1 and SpCas9	NGS-F1	ATCGGGAAGCTGAAGATCTGCCGACGCGGCGAGCTTCCG
	NGS-F2	ATCGGGAAGCTGAAGGCTCTACGACGCGGCGAGCTTCCG
	NGS-F3	ATCGGGAAGCTGAAGCGAGATCGACGCGGCGAGCTTCCG
	NGS-F4	ATCGGGAAGCTGAAGTAGACGCGACGCGGCGAGCTTCCG
	NGS-R	ATCCGACGGTAGTGTTCATGCGGTACTGCACCGGGT
Primary PCR waxy (SpCas12f1 and SpCas9)	NGS-F1	ATCGGGAAGCTGAAGATCTGCGACGTCGTGTTCGTCTGCAAC
	NGS-F2	ATCGGGAAGCTGAAGGCTCTAGACGTCGTGTTCGTCTGCAAC
	NGS-F3	ATCGGGAAGCTGAAGCGAGATGACGTCGTGTTCGTCTGCAAC
	NGS-F4	ATCGGGAAGCTGAAGTAGACGGACGTCGTGTTCGTCTGCAAC
	NGS-R	ATCCGACGGTAGTGTTCTGGTAGGTAGTACGTGAAGATGGT
Universal secondary PCR forward primer		AATGATACGGCGACCACCGAGATCTACACATACGAGATCCGTAATCGGGAAGCTGAAG
Universal secondary PCR reverse primer ("N" indicates variable 8 bp index sequence for sample deconvolution)		CAAGCAGAAGACGGCATACGAGATNNNNNNNNACACGCACGATCCGACGGTAGTGT
Amplicon sequencing primer		CATACGAGATCCGTAATCGGGAAGCTGAAG

Index sequencing primer	ACACTACCGTCGGATCGTGCGTGT

Supplementary Table 6 | gRNA targets used in human and maize cell editing.

**Protein**	**Target Name**	**Target sequence (5'-3')**
**For genome editing in human cells**		
AsCas12a	Non-target (NT)	AGTTGACCCAACGTCGCCGGCGT
	VEGFA3	GCCAGAGCCGGGGTGTGCAGACG
	VEGFA2	AGTGCGACGCCGCGAGCCCCGAC
	DNMT1	GCTCAGCAGGCACCTGCCTCAGC
AsCas12f1	Non-target (NT)	AGTTGACCCAACGTCGCCGG
	VEGFA3	GCCAGAGCCGGGGTGTGCAG
	VEGFA2	GGGGTGACCGCCGGAGCGCG
	DNMT1	GCTCAGCAGGCACCTGCCTC
SpCas12f1	Non-target (NT)	AGTTGACCCAACGTCGCCGG
	VEGFA3	CCTCTTTAGCCAGAGCCGGG
	VEGFA2	AGTGCGACGCCGCGAGCCCC
	DNMT1	CCTCACTCCTGCTCGGTGAA
**For genome editing in maize cells**		
SpCas12f1	*ms26*	AAGTTCACGGCGTTCCAGGC
	waxy	AGTTCAGAGAAGGCAACCTT
SpCas9	*ms26*	AAGTTCACGGCGTTCCAGGC
	waxy	GGCATCTACAGGGACGCAA
