# Supplementary material for: Miniature type V-F CRISPR-Cas nucleases enable targeted DNA modification in cells
Source: Nat Commun. 2021 Oct 26;12:6191. doi: 10.1038/s41467-021-26469-4 (PMC8548392; doi:10.1038/s41467-021-26469-4)
Supplement: Supplementary file 6 — Description of Additional Supplementary Files [file 41467_2021_26469_MOESM6_ESM.pdf]

Title: Supplementary Data 1
Description: Percentage of targeted mutation(s) recovered in T0 plants at the *waxy* site. The percentage of edited reads recovered at the *waxy* target site in each regenerated plant is shown associated with each treatment, 28°C and either one (1x) or three (3x) 4 hr long heat treatments at 45°C. SpCas12f1 expression cassettes biolistically transformed into immature maize embryos are also listed along with plant identifier (ID) and Illumina sequencing LIMs ID.

Title: Supplementary Data 2
Description: Percentage of targeted mutation(s) recovered in T0 plants at the *ms26* site. The percentage of edited reads recovered at the *ms26* target site in each regenerated plant is shown associated with each treatment, 28°C and either one (1x) or three (3x) 4 hr long heat treatments at 45°C. SpCas12f1 expression cassettes biolistically transformed into immature maize embryos are also listed along with plant identifier (ID) and Illumina sequencing LIMs ID.

Title: Supplementary Data 3
Description: Predicted and evaluated SpCas12f1 *ms26* and *waxy* corn off-target sites. Cas-OFFinder predicted (p) sites and targets with sequence coverage (c) are shown in adjacent columns for each SNP and bulge combination associated with each treatment. Successfully covered sites contained sequence data for at least three T0 plants. Percent coverage for sites with up to three SNPs and zero bulges (columns B-U) was 100% while coverage for targets with greater than three SNPs (columns V-AE) ranged from 72-84% for each treatment (column AF) or 72-82% when average across target site (column AG).

Title: Supplementary Data 4
Description: Evaluation of SpCas12f1 off-target editing in *ms26* and *waxy* edited T0 corn plants. Each treatment, 1x 45°C and 3x 45°C, and target site, *ms26* and *waxy*, has its own tab. On each tab, the DNA and RNA sequence (both associated with the TTC protospacer adjacent motif (PAM) for SpCas12f1) of the intended and Cas-OFFinder predicted off-target sites are shown (columns A and B). The number of differences (mismatches (SNPs), bulges and mismatches plus bulges (MMB)) from the on-target are also listed (columns C-E). Additionally, MIPs and targeted sequencing (TargetedSeq) information including MIPs genomic hybridization sequences (LigArm and ExtArm), forward (FP) and reverse (RP) primers and amplicon sequence (AmpliconSeq) and length (AmpliconLen) are included (columns G-N). The number of T0 plants analyzed by MIPs and targeting sequencing as well as the editing result can be found in columns O-R.
